# ABLIM1, a novel ubiquitin E3 ligase, promotes growth and metastasis of colorectal cancer through targeting IĸBα ubiquitination and activating NF-ĸB signaling

**DOI:** 10.1038/s41418-024-01256-y

**Published:** 2024-01-16

**Authors:** Ying He, Qian Shi, Yuhang Ling, Huihui Guo, Yi Fei, Ruoyu Wu, Chengwu Tang, Xilin Zhang, Linhua Yao

**Affiliations:** 1 Central Laboratory, First Affiliated Hospital of Huzhou University, Huzhou, 313000 Zhejiang China; 2 Huzhou Key Laboratory of Translational Medicine, First People’s Hospital of Huzhou, Huzhou, 313000 Zhejiang China; 3 Department of Colorectal Surgery, First Affiliated Hospital of Huzhou University, Huzhou, 313000 Zhejiang China; 4 Department of Gastroenterology, First Affiliated Hospital of Huzhou University, Huzhou, 313000 Zhejiang China

**Keywords:** Metastasis, Ubiquitin ligases, Oncogenes

## Abstract

Actin-binding LIM protein 1 (ABLIM1), a member of the LIM-domain protein family, has been reported as a suppressor in several tumors whereas its role in colorectal cancer (CRC) remains unknown. In this study, we find that ABLIM1 is up-regulated in CRC patients and high levels of ABLIM1 predict short disease-free survival time. Knock-down of ABLIM1 in CRC cell lines by lenti-virus leads to inhibited cell proliferation, migration, and invasion capabilities in vitro and impaired growth of tumor xenografts and liver metastasis lesions in vivo, while ABLIM1 overexpression accelerates tumor growth and invasion in vitro. Mechanistically, we uncover that ABLIM1 activates the NF-ĸB/CCL-20 signaling through modulating IĸBα ubiquitination and proteasomal-mediated degradation. Further co-immunoprecipitation, in vivo and in vitro ubiquitination assays reveal ABLIM1 as a novel ubiquitin E3 ligase binding to IĸBα. Interestingly, The E3 ligase catalysis activity of ABLIM1 depends on its 402–778aa rather than its LIM domains and its interaction with IĸBα relies on the HP domain. Our findings delineate the oncogenic role of ABLIM1 in CRC progression and reveal it as a novel E3 ligase targeting IĸBα, providing new insights into the regulation of NF-ĸB signaling in tumors.

## Introduction

Colorectal cancer (CRC) is the third most diagnosed tumor and the second cause of cancer-related mortality worldwide [1]. Metastasis is the primary cause of CRC-related death, with liver metastasis accounting for the most cases. As previously reported, about 25% of CRC patients have liver metastasis at first diagnosis and nearly 50% of the cases develop liver metastasis along with the development of CRC [2]. Although remarkable advances have been achieved in cancer diagnosis and treatment, the existing treatment strategies for metastatic CRC remain limited and unsatisfactory, especially in consideration of the increasing tide of CRC in younger adults and transitioning countries [1, 3]. Hence, there is a pressing need for clarifying the molecular mechanism of CRC progression and metastasis.

NF-ĸB signaling is a crucial regulator of cell growth, inflammation, metastasis, and drug resistance in CRC [4] and multiple other cancers [5]. NF-ĸB complex consists of five members, including RelA (p65), RelB, c-Rel, NF-ĸB1 (p50), and NF-ĸB2 (p52). At the quiescent state, they are sequestered in the cell cytoplasm by IĸB proteins. A canonical mechanism of NF-ĸB activation is that IĸB, like IĸBα, experiences phosphorylation, ubiquitination, subsequent proteasome-mediated degradation, and thus p65/p50 dimer translocates to the nucleus to induce gene expressions [6], including inflammatory cytokines, chemokines, and proliferation-associated genes [4]. Increasing evidence supports the over-activation of NF-ĸB as a hallmark of CRC [4] and thus inhibitors targeting NF-ĸB are being investigated as promising therapeutic approaches for CRC [7].

Protein ubiquitination represents a prominent post-translation modification controlling the activity of proteins, such as IĸBα [8] and p65 [6]. For IĸBα, one well-studied mechanism of IĸBα ubiquitination is mediated by the SCF (Skp, Cullin, F-box) E3 ligase complex [8], whereas the ubiquitination regulation of IĸBα is far from clarified. More and more novel E3 ligases [9–11] have been found to take a part in the stability and degradation modulation of IĸBα. For example, TRIM22 [10] and a RING finger-domain E3 ligase RBBP6 [11] have been elucidated to bind and ubiquitinylate IĸBα, influencing NF-ĸB signaling activity in glioblastoma and CRC.

Actin-binding LIM protein 1 (ABLIM1), which contains 4 LIM domains, a coiled-coil domain, and HP domain, belongs to a large LIM-domain protein family. LIM-domain family proteins are thought to provide protein-protein interaction scaffolds and mounting binding partners have been identified in recent years [12**–**14]. Structurally, LIM-domain proteins contain one or more Zn-finger motifs that are like really interesting new gene (RING) [15] and thus are predicted to possess E3 ligase activities [12], though the functions of most of them in cancers, including ABLIM1, remain unclear.

This is the first study, to the best of our knowledge, to reveal ABLIM1 as a novel E3 ligase targeting IĸBα and its aberrantly high expression promotes CRC growth and metastasis in vitro and in vivo through activating NF-ĸB p65/CCL20 signaling. Notably, the E3 ligase activity of ABLIM1 depends on its 402–778aa rather than its LIM domains and its interaction with IĸBα relies on the HP domain, which is unlike other LIM-domain proteins that possess E3 ligase activities. These findings indicate ABLIM1 as a new E3 ligase whose structure might be unlike any other traditional E3 ligases. Moreover, we reveal the oncogenic role of ABLIM1 in CRC growth and metastasis by modulating IĸBα ubiquitination and degradation, providing novel insights into the complex regulation mechanism of NF-ĸB signal in tumors.

## Materials and methods

### Reagents, antibodies, and plasmids

Cycloheximide (CHX) and MG132 were purchased from MedChemExpress. PS341 was from TargetMol (USA). BAY 11–7082, G418, Puromycin, and CCK-8 were from Beyotime Biotechnology (Shanghai, China). Matrigel was obtained from Corning (New York, USA) and Ni-NTA agarose was from Invitrogen (California, USA). CCL-20 enzyme-linked immunosorbent assay (ELISA) kit was from Boster (Wuhan, China) and E3 ligase ubiquitinylation assay kit was from Enzo Life Sciences (USA). Primary antibodies used were ABLIM1 (A302-237A-T, Bethyl), p-IĸBα S36 (ab133462, Abcam), IĸBα (4814, Cell Signaling Technology), NF-ĸB p65 (8242, Cell Signaling Technology), p-p65 S536 (3033, Cell Signaling Technology), LaminB1 (66095-1-Ig, Proteintech), ubiquitin (10201-2-AP, 1:1000, Proteintech), CCL20 (A1756, Abclonal), HA (3724, Cell Signaling Technology), Flag (AF5051, Beyotime), Ki-67 (27309-1-Ig, Proteintech), β-actin (M1210-2, HuaBio), and GAPDH (60004-1-Ig, Proteintech). Human DNA fragments of RELA and ubiquitin obtained from PCR were cloned into pcDNA3.1 to generate constructs with a Flag tag or His tag, respectively. The plasmids containing 1-216aa, 1-401aa, 402-778aa, 402-778aa mutants (coiled-coil domain deleted or HP domain deleted), 1-709aa (∆HP, an E3 ligase dead mutant form of ABLIM1), shRNA-resistant ABLIM1 (ABLIM1^shMut^), or wide type human ABLIM1 were generated with an N-terminal 3 × Flag tag, respectively. Constructs containing 1-109aa, 110-181aa, 182-317aa, wide type, or non-phosphorylation mutant (S32/36 A) of IĸBα were established with an N-terminal HA tag, respectively. Lenti-viruses containing ABLIM1 shRNA (shRNA#1 target sequence, CCCTGAAGTGTTTCGGGAAAT; shRNA#2 target sequence, GCCACTTTATTATCATGCTTT) or control scramble shRNA (Target sequence, CCTAAGGTTAAGTCGCCCTCG) were purchased from TsingKe BiotechnologyCo., Ltd.

### Patient specimens

One-hundred and forty-four pairs of paraffin-embedded colorectal tumors and adjacent normal mucosae were retrospectively collected from CRC patients who underwent surgical resection of primary CRC in the First People’s Hospital of Huzhou between Jan 2018 and Oct 2019. After sectioning and HE staining confirmation of the tissue pathological types, the 144 pairs of tissues were constructed into 2 tissue microarray blocks at Haoke Biotechnology Co. Ltd. (Hangzhou, China). Forty-four freshly frozen colorectal tumor tissues and adjacent normal mucosae were collected between April 2020 and December 2022. This study was approved by the Ethics Committee of the First People’s Hospital of Huzhou (No. 2020KYLL002). Informed consent was obtained from all patients.

### Cell culture, plasmid transfection, and virus infection

Human colon carcinoma cell lines HCT 116 (Cat#TCHu99), RKO (Cat#TCHu116), Lovo (Cat#SCSP-514), SW480 (Cat#SCSP-5033), and SW620 (Cat#TCHu101) were obtained from National Collection of Authenticated Cell Cultures of China (Shanghai, China). Human normal colon epithelial cell line, HCoEpic (Cat#MZ-0806), was purchased from MingzhouBio (Ningbo, China). Human embryonic kidney 293 T cell line (Cat#CL-0005) was obtained from Procell Life Science (Wuhan, China). All cell lines were authenticated by short tandem repeat profiling and mycoplasma examinations were done using Mycoplasma Detection Kit (Beyotime). HCT 116 was cultured in Mccoy’s 5 A medium and the other cell lines were cultured in DMEM medium, supplemented with 10% FBS (Gibco). For plasmid transfection, cultured HCT116, SW620, RKO, or 293 T cells were transfected with indicated plasmid constructs using Lipofectamine 2000 (1662298, Invitrogen) according to the manufacturer’s instructions. To obtain the stable cell line overexpressing ABLIM1, HCT 116 cells transfected with pcDNA3.1-ABLIM1-3×Flag were selected by G418 (400 μg/mL) for 2 weeks. For ABLIM1 knockdown, HCT 116, RKO, and SW620 cells were infected with lentivirus particles containing shRNA#1, shRNA#2, or scramble control at the MOI of 5, 10, and 30 for 24 h, respectively. Three days after infection, the knockdown efficiency was assessed by immunoblots to screen the optimal infection condition. Stable cells were generated by puromycin selection (1 μg/mL) for 2 weeks.

### Cell proliferation, migration, and invasion assays

For cell viability assay using the Real-time cell analysis (RTCA) xCELLigence^®^ system (ACEA Biosciences, USA), manipulated HCT 116, RKO, or SW620 cells were seeded in E-16 plates (2 × 10^4^ cells/well), the cell indexes were recorded during the following 3 days. For cell viability using the CCK-8 kit, cells were seeded into 96-well plates and the absorbances of 450 nm at indicated time points were assayed on the SpectraMax190 microplate reader after incubation with CCK-8 reagent for 1 h. To further evaluate the cell colony formation, manipulated HCT 116 or RKO cells were seeded into 6-well plates at a density of 100,000 cells/well and then cultured in the incubator with 5% CO_2_ at 37 °C for 7 days. Cell colonies were then fixed with 4% polyformaldehyde (PFA) and stained with 1% (w/v) crystal violet solution.

The RTCA and CIM-16 plates were used to assess the cell migration and invasion capacity according to the instructions [16]. Briefly, manipulated HCT 116, RKO, or SW620 cells were seeded into CIM-16 plates with or without pre-coated matrigel (dilution, 1:10 for HCT 116 and RKO, 1:20 for SW620) and cell indexes were monitored by the RTCA for 72 h. The migration and invasion abilities of cells were further examined by using Transwell chambers (Corning, USA). Transwell chambers were fixed with 4% PFA at the indicated time and migrated and invaded cells were stained with 1% crystal violet solution. Transwell assays were performed in triplicate and repeated at least two times.

### Animal experiments

The animal experiments were conducted under the approval of the Ethics Committee of the First People’s Hospital of Huzhou (No. 2020KYLL002). Six-week-old male BALB/c nude mice were raised in a specific pathogen-free environment. “*E* value” analysis based on the “resource equation” method was used to estimate the sample size in animal experiments. For the tumor xenograft experiment, stable shABLIM1-KD HCT 116 cells or scramble shRNA control cells were inoculated subcutaneously into the flanks of nude mice (5 × 10^6^ cells/100 μL/mouse, *n* = 6 per group). Tumor sizes were measured every 2–3 days with a vernier caliper and the tumor volumes were calculated according to the formula *V* = 0.5 × Length × Width^2^. To establish the liver metastasis model of CRC, stable shABLIM1 SW620 cells or scramble shRNA control cells were injected into the hepatic portal veins of nude mice as previously reported [17]. Nude mice were randomly divided into the shABLIM1 group or control group for xenografts and metastasis models. The investigators were not blinded to the group allocation. After sacrifice, subcutaneous tumor xenografts and livers in the metastatic model were excised from the mice and fixed in 4% FPA for immunohistochemical experiments.

### RNA-sequencing and enrichment analysis

Cultured stable shABLIM1 HCT 116 cells or scramble shRNA control cells were rinsed with cold PBS, digested with trypsin, and then resuspended in TRIZOL buffer. Transcriptome sequencing was performed on the Hi-Seq Illumina platform in Tsingke Biotechnology Co., Ltd. Gene expression data were analyzed, and differentially expressed genes (DEGs) were identified according to the standard comparison mode with multiple testing corrections (fold change > 1.5, P_adj_ < 0.05). All DEGs were given in the supplementary Table S1. Kyoto Encyclopedia of Genes and Genomes (KEGG) annotation and pathway enrichment was conducted and visualized using R software by Tsingke Biotechnology Co., Ltd.

### Immunoblots and co-immunoprecipitation (co-IP)

Cells were lysed using RIPA strong buffer (Beyotime, Shanghai, China) supplemented with protease and phosphatase inhibitor cocktails (Cwbio, Taizhou, China) and 1 mmol/L PMSF on ice for 30 min to test the total cell proteins. For examination of separate cytoplasmic and nuclear proteins, cytoplasmic and nuclear cell contents were extracted following the instructions of Nuclear and Cytoplasmic Protein Extraction kit (Beyotime). In co-IP assay, cells were lysed with Nonidet P-40 buffer containing protease and phosphatase inhibitor and then the obtained lysis supernatants were incubated with primary anti-ABLIM1, anti-IĸBα, or anti-HA antibody at 4 °C overnight. The next day, protein A beads or protein G beads were added and incubated for 3 h. After washing with PBS-T 3 times, the beads were boiled with loading buffer to elute the interacted components. The total or separate cell contents and IP precipitates were separated by SDS-PAGE, respectively. Proteins were transferred onto polyvinylidene difluoride membranes, which were blocked with 5% BSA for 1 h and then incubated with corresponding primary antibodies. HRP-conjugated secondary antibodies were added and proteins were examined by the chemiluminescence imaging system (Tanon 5200, Shanghai, China). All original western blots are available in the Supplementary Material.

### Immunohistochemistry (IHC) and immunofluorescence (IF)

Paraffin-embedded tissue microarray blocks or mice samples were sectioned into 4 μm slides for immunohistochemistry. Slides were stained for ABLIM1 (dilution, 1:200), p65 (dilution, 1:400), Ki-67 (dilution, 1:1000), or CCL-20 (dilution, 1:200) and then counterstained with hematoxylin according to the standard procedure as previously reported [18, 19]. For analysis of the stained slides, two pathologists blinded to the sample identity scored the immunohistochemical images using the H-score rules. According to the percentage of positively stained cells, samples were scored as 0 (< 10%), 1 (10–25%), 2 (26–50%), 3 (51–75%), or 4 points (> 75%). Based on the staining intensity, samples were scored as 0 (negative), 1+ (light brown), 2+ (brown), or 3+ (dark brown). H-score = 1× (% cells 1+) + 2× (% cells 2+) + 3× (% cells 3+).

Stable shABLIM1 HCT 116 cells, RKO cells, and control cells were cultured in 6-well plates containing cell slides for 2 days and then fixed with 4% PFA. After washing with PBS, fixed cells were blocked with 5% normal goat serum containing 0.3% Triton X-100 for 60 min, followed by primary anti-p65 antibody (dilution, 1:200) incubation at 4 °C overnight. The next day, cells were incubated with donkey anti-rabbit secondary antibody conjugated with Alexa-555 for 60 min and the nuclear staining was done using DAPI. Finally, digital IF images were obtained using a TMA scanner (3D Histech, Hungary), and p65 expression differences in nucleus and cytoplasm between shABLIM1 and control cell groups were observed.

### RNA isolation and quantitative real-time PCR (qRT-PCR)

For tissue samples, total RNA was extracted with TRIZOL after homogenization with a homogenizer (Shanghai, China). For cell samples, RNA was obtained using the RNA-Quick Purification Kit (Yishan Biotechnology, Shanghai, China). Reverse transcription was conducted using a PrimeScript kit (Takara, Beijing, China). Real-time PCR was performed on the ABI 7500 instrument using SYBR green mix (Cwbio) following the manufacturer’s instructions. Primers used were listed in the supplementary Table S2. The 18 S ribosomal RNA was used as the internal control. Relative gene expression levels were calculated using the 2^-^^ΔΔCt^ method.

### ELISA

shABLIM1 or ABLIM1 overexpressed HCT 116 cells, RKO cells, SW620 cells, and control cells were cultured in 24-well plates until reaching confluence. Cell culture media were changed into phenol red-free media and cultured for another 24 h. Cell culture supernatants were collected after centrifugation at 800 rpm for 5 min. ELISA detection of human CCL-20 in those cell culture supernatants was conducted following the manufacturer’s instructions. Samples or standard controls were added into the 96-well plate which was pre-coated with antibody specific to CCL-20 and incubated at 37 °C for 2.5 h. After extensive washing, the biotinylated detection antibody of CCL-20 was added for 1-h incubation. For signal development, the plate was incubated with HRP-conjugated secondary antibody and then tetramethyl-benzidine (TMB) substrate, followed by color stopping with a sulfuric acid solution. Optical densities at 450 nm were measured on the SpectraMax190 microplate reader. The standard curve was determined using regression analysis and CCL-20 concentrations in cell supernatants were calculated accordingly.

### In vivo and in vitro ubiquitination assay, auto-ubiquitination assay

For in vivo ubiquitination assay, HCT116 cells transfected with his-tagged ubiquitin, and ABLIM1 or empty vector were cultured for 48 h and treated with MG132 (20 μM, 6 h) before harvest. Cell samples were immediately frozen using liquid nitrogen for storage. His-tagged proteins and interacted components were purified using Ni-NTA beads (Invitrogen, USA) as previously reported [6]. Samples were lyzed by 6 M guanidinium-HCl in 0.1 M Na_2_HPO_4_/NaH_2_PO_4_ buffer (pH = 8.0) and then the lysates were passed through 18 G needles 5 times to shear the DNA and reduce the viscosity. Lysate supernatants were incubated with Ni-NTA beads at 4 °C overnight. The next day, the Ni-NTA beads were washed 5 times, once by lysis buffer, twice by lysis buffer diluted in 25 mM Tris-HCL/20 mM imidazole (dilution, 1:4), and twice by 25 mM Tris-HCL/20 mM imidazole. Finally, the purified proteins were eluted by boiling the beads with 200 mM imidazole and then examined by immunoblotting.

For in vitro ubiquitination assay, the lysate from ABLIM1-overexpressed cells was immunoprecipitated by anti-ABLIM1 antibody, and then the beads were incubated with E1, E2, recombinant IĸBα (Abclonal, Cat#RP00032), ubiquitin, DTT, and ubiquitination buffer at 37 °C for 6 h in accordance with the protocol of the ubiquitination kit (Enzo Life Sciences, Cat#BML-UW9920). After incubation, non-reducing gel loading buffer was added and the IĸBα ubiquitination products were analyzed by immunoblotting.

In auto-ubiquitination assay, recombinant proteins of human ABLIM1 1-401aa (Abnova, Cat#ABN-H00003983), 376-778aa (Solarbio, Cat#02608) were used. The purity of the proteins was validated by SDS-PAGE. According to instructions from the manufacturer (Abcam, Cat# ab139469), recombinant ABLIM1 1-401aa, 376-778aa, and negative PBS control were incubated with 20 × E1, 20 × UbcH5a, 10 × ubiquitin, 20 × Mg-ATP, 50 mM DTT in 10 × E3 ligase buffer at 37 °C for 2 h, respectively. Then, they were boiled with loading buffer, followed by immunoblotting detection.

### Dual-luciferase reporter assay

The NF-κB binding-element reporter plasmid, pGL6-NFκB-TA-luc (Beyotime), was used to monitor the activity of NF-κB signaling and pRenilla-TK plasmid was used as the internal control to normalize the transfection efficiency. Different constructs (ABLIM1 full-length, 402-778aa, 1-401aa, IκBα S32/36 A phosphorylation mutant, or empty vector) were co-transfected with pGL6-NFκB-TA-luc and pRenilla-TK into HCT116 or RKO or SW620 cells as indicated. Forty-eight hours after transfection, firefly and renilla luciferase activities were determined using the Dual-luciferase kit (Promega) on the IVIS Lumina LT Series Ш in vivo imaging system (Perkin Elmer). The relative luciferase activities of firefly to renilla were calculated and average results from three biological replicates were used to represent the NF-κB binding-element reporter activities.

### Database analyses

For survival analysis, patient survival data and mRNA expression data of two datasets (TCGA PanCancer Atlas, TCGA Firehose Legacy) were downloaded from the CBioPortal database for cancer genomics (http://www.cbioportal.org/). The optimal cutoff determination and following survival analysis were conducted using an online bioinformatic platform, Sangerbox (http://www.sangerbox.com/tool) [20]. Correlation analysis between RELA and ABLIM1 was done in the GEPIA2 database by using TCGA COAD, READ, and normal counterpart data.

### Statistical analysis

Data were shown as Mean ± SD of at least three biological replicates unless special description. Student’s *t*-test was used for normally distributed samples (*n* > 3); otherwise, nonparametric test was used. Two-sided *p* < 0.05 was considered statistical significance. Statistical analysis and figure visualization were performed in SPSS and GraphPad Prism 8.

## Results

### ABLIM1 is highly expressed in CRCs and augmented expression correlates with poor disease-free survival

**Fig. 1:**
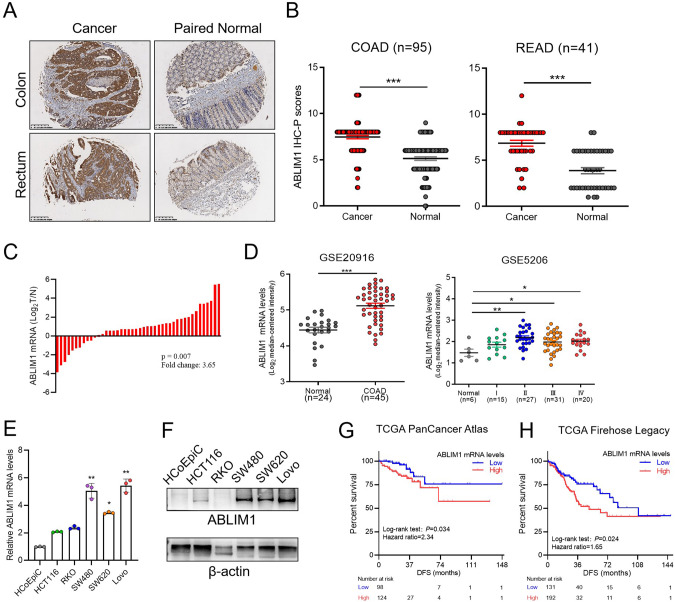
ABLIM1 is aberrantly overexpressed in CRCs and augmented expression correlates with poor disease-free survival. **A** Representative ABLIM1 immunohistochemistry staining images of the CRC samples and adjacent normal counterparts in a tissue microarray. **B** Semiquantitative analysis results of immunohistochemistry staining images based on the H-score method. Data were shown as Mean ± SEM. ^***^P < 0.001 by paired student’s *t*-test. **C** Relative mRNA levels of ABLIM1 in 44 cases of freshly frozen CRC samples and their paired normal control mucosae. *P* = 0.007 determined by paired *t*-test. **D** ABLIM1 mRNA expressions in two datasets (GSE20916 and GSE5206) of the GEO database. Data were shown as Mean ± SEM. Unpaired student’s *t* test: ^*^P < 0.05^; **^P < 0.01; ^*^^**^P < 0.001. **E** Relative mRNA levels of ABLIM1 in a normal human colon epithelial cell line, HCoEpic, and 5 human CRC cell lines, including HCT116, RKO, SW480, SW620, and Lovo. *N* = 3 for each cell line. Kruskal–Wallis test. **F** ABLIM1 protein levels in HCoEpic and 5 human CRC cell lines were assayed by immunoblotting. Kaplan-Meier disease-free survival curves stratified by ABLIM1 expression levels using data downloaded from the CBioPortal database (**G**, TCGA PanCancer Atlas; **H**, TCGA Firehose Legacy). **G** *P* = 0.034, hazard ratio = 2.34 [1.04–5.33, 95% confidence interval (CI)]. **H**, *P* = 0.024, hazard ratio = 1.65 [1.08–2.50, 95% CI].

To grasp the expression profile of ABLIM1 in human colorectal cancer, we investigated its mRNA and protein expression levels in freshly frozen CRC samples, paraffin-embedded tissue microarray, human colorectal cell lines, and GEO database. The immunohistochemistry staining images and semiquantitative analysis revealed that ABLIM1 was aberrantly overexpressed in colon and rectum tumors as compared with the adjacent normal counterparts (Fig. 1A, B). Enhanced ABLIM1 mRNA expression in colorectal cancers was observed in both freshly frozen CRC samples in our cohort and the GEO datasets (GSE20916 and GSE5206) (Fig. 1C, D). Data in the GEPIA 2 database indicated ABLIM1 expression was reduced in 17 types of tumors but enhanced in colorectal tumor and 3 other tumors (Figure S1A). ABLIM1 was up-regulated in multiple colon carcinoma cell lines, including HCT116, SW480, SW620, and Lovo, when compared with the normal colon epithelial cell line, HCoEpic (Fig. 1E, F). Interestingly, ABLIM1 has been previously reported to be down-regulated and act as a candidate tumor suppressor in glioblastoma [21] and melanoma [22], whereas its expression pattern and role remain unknown in colorectal cancers. The completely different expression profile we found here suggests a distinct role and functional mechanism of ABLIM1 in CRCs. By querying the CBioPortal database for cancer genomics, we found that augmented expression ABLIM1 significantly predicted short disease-free survival time in CRC patients in two independent studies (TCGA PanCancer Atlas, *P* = 0.034, hazard ratio = 2.34; TCGA Firehose Legacy, *P* = 0.024, hazard ratio = 1.65) (Fig. 1G, H), though no statistical significance was observed in overall survival analysis (Fig. S1B, C), which indicates ABLIM1 upregulation as an independent predictor of disease relapse. Taken together, these findings suggest ABLIM1 may closely associate with CRC relapse and play a role in tumor malignant progression.

### ABLIM1 acts as an oncogene promoting CRC growth and metastasis in vitro and in vivo

**Fig. 2:**
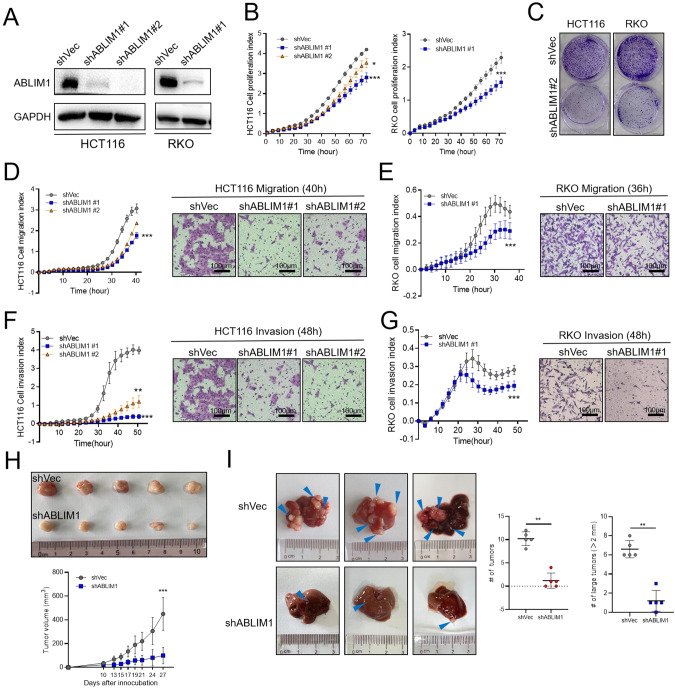
ABLIM1 silence inhibits CRC growth and metastasis in vitro and in vivo. **A** Western blot validation of the knock-down efficiency of lenti-virus shABLIM1#1 and shABLIM1#2 in HCT116 and RKO cells. **B** Cell proliferation indexes of stable shABLIM1 HCT116, RKO, and control shVec cells detected by Real-time cell analysis (RTCA) xCELLigence^®^ system. For HCT116, *n* = 3 or 4 for each group. For RKO, *n* = 5 for each group. **C** HCT116 and RKO cell colonies formed in 6-well plates after 10-day culture were visualized by crystal violet staining. Cell migration assays using the RTCA system and Transwell chambers in HCT116 cells **D** and RKO cells **E**. For HCT116, *n* = 3 or 4 for each group. For RKO, *n* = 5 or 6 for each group. Cell invasion assays using the RTCA system and Transwell chambers in HCT116 cells **F** and RKO cells **G**. For HCT116, *n* = 3 or 4 for each group. For RKO, *n* = 7 for each group. For *n* > 3, paired student’s *t*-test; For *n* = 3, Friedman test in **B–G**. **H** Effect of ABLIM1 knockdown on CRC xenograft growth in nude mice. Stable shVec or shABLIM1 HCT116 cells were subcutaneously injected into nude mice (*n* = 6) and the tumor xenografts were photographed after sacrificing the mice on day 27 after inoculation. Unpaired student’s *t*-test. **I** Effect of ABLIM1 knockdown on CRC liver metastasis in nude mice. Stable shVec or shABLIM1 SW620 cells were injected into the hepatic portal veins of nude mice (*n* = 5) and the liver metastasis nodes were photographed after sacrificing the mice on day 25. Blue triangles indicate the tumor nodes in livers. Mann Whitney test. ^*^P < 0.05^; **^P < 0.01; ^***^P < 0.001.

The biological function of ABLIM1 was studied using various in vitro cell experiments and in vivo tumor xenografts and metastasis models. In consideration of the different genetic mutations HCT116 (*KRAS*^G13D^*PIK3CA*^H1047R^), RKO (*BRAF*^G13D^*PIK3CA*^H1047R^), and SW620 (*KRAS*^G12V^*TP53*^R273H; P309S^) harbored [23], these three cell lines were chosen in our experiments to make the findings more reliable and representative. We screened 3 lenti-shRNA viruses and identified two efficient ones, named shRNA #1 and shRNA#2. In stable HCT116 and RKO cells infected with lenti-virus, ABLIM1 could be successfully silenced by shRNA#1 and shRNA#2 (Fig. 2A). By using the RTCA system, the real-time indexes of cell proliferation, migration, and invasion could be recorded and visualized clearly. After ABLIM1 depletion, the proliferation capacity of RKO and HCT116 during 3 days were both markedly inhibited (Fig. 2B). Since cells transduced with shRNA#1 showed more obvious growth retardation, we further used them in cell colony formation assay to evaluate the long-term effect of ABLIM1 depletion on cell proliferation. The results in Fig. 2C showed that ABLIM1 knockdown could repress colony formation significantly. In cell migration assays using RTCA and transwell chambers, migration of HCT116 cells was impeded after ABLIM1 knockdown (Fig. 2D). Consistent differences were observed between ABLIM1-silenced RKO and control cells in both RTCA cell migration curves and crystal violet-stained images of migrated cells in transwell chambers (Fig. 2E). To investigate the cell invasion changes, matrigel solution was added into the RTCA system and transwell chambers (40 μL/well), respectively. The RTCA invasion curve and transwell images indicated that both the cell invasion speed and invaded cell numbers of HCT116 and RKO were decreased markedly after ABLIM1 silence (Fig. 2F, G). To exclude the off-target effect, we constructed a synonymous mutant ABLIM1 of shRNA1 target sequence (ABLIM1^shMut^, a shRNA-resistant form of ABLIM1) and overexpressed it in shABLIM1 #1-treated HCT 116 cells to observe the rescue influence. As shown in Fig. S2, ABLIM1^shMut^ overexpression specifically reversed the inhibitory effect of shABLIM1 on cell proliferation, migration, and invasion, implying the shRNA is directly linked to the phenotypes we observed. Thus, it is reasonable to suspect ABLIM1 as an oncogene in CRC, which could promote tumor cell proliferation, migration, and invasion. Furthermore, we explored the role of ABLIM1 in vivo. Stably manipulated HCT116 and SW620 cell lines were obtained using lenti-ABLIM1 shRNA #1 or control viruses. In the tumor xenograft experiment, ABLIM1 knockdown in HCT 116 cells remarkably slowed down the subcutaneous tumor growth (Fig. 2H). Especially, ABLIM1 knockdown dramatically reduced the number and size of metastasis nodules in nude mouse livers (Fig. 2I), and corresponding HE images under microscopy also displayed the significant differences of tumor loci area in livers before and after ABLIM1 knockdown (Supplementary Fig. S3B), suggesting the crucial role of ABLIM1 in CRC invasion and metastasis.

**Fig. 3:**
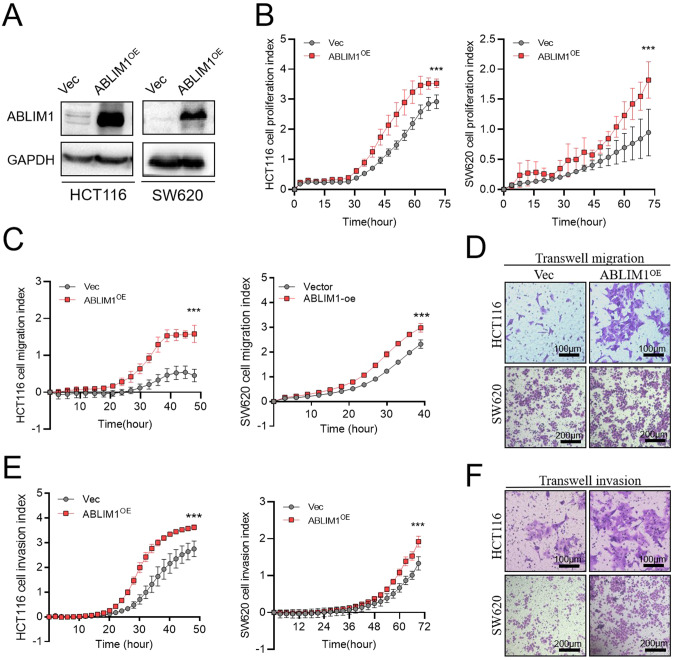
ABLIM1 overexpression promotes CRC growth, migration, and invasion. **A** Western blot validation of the ABLIM1 overexpression efficiency in HCT116 and SW620 cells. **B** Cell proliferation indexes of HCT116 and SW620 cells transfected with pcDNA3.1-ABLIM1 or empty vectors were recorded by RTCA system. For HCT116, *n* = 4 for each group. For SW620, *n* = 3 for each group. **C** Cell migration indexes of HCT116 and SW620 cells detected by the RTCA system. For HCT116, *n* = 8 for each group. For SW620, *n* = 4 for each group. **D** For HCT116 and SW620 cells transfected with pcDNA3.1-ABLIM1 or empty vectors, the migrated cells in Transwell chambers were stained by 1% crystal violet solution. **E** Cell invasion indexes of HCT116 and SW620 cells transfected with pcDNA3.1-ABLIM1 or empty vectors detected by the RTCA system. Matrigel dilution ratios: HCT116, 1:10; SW620, 1:20. *n* = 8 for each group. **F** Cell invasion results of HCT116 and SW620 cells transfected with pcDNA3.1-ABLIM1 or empty vectors were visualized by crystal violet staining. For *n* > 3, paired student’s *t*-test; For *n* = 3, Friedman test. ^*^P < 0.05^; **^P < 0.01; ^***^P < 0.001.

To further validate the tumor-promoting role of ABLIM1, ABLIM1 was overexpressed in two colon cancer cell lines (Fig. 3A) by plasmid and thereafter its influence on tumor cell proliferation, migration, and invasion was assessed. As shown in RTCA proliferation curves, ABLIM1 overexpression accelerated HCT116 and SW620 cell growth (Fig. 3B). In line with the results in Fig. 2, ABLIM1 overexpression contributed to tumor migration and invasion in both RTCA system and transwell assays (Fig. 3C–F). Collectively, these functional experiments demonstrated that ABLIM1 serves as an oncogene, promoting CRC growth and metastasis in vitro and in vivo.

### ABLIM1 modulates IĸBα/NF-κB/CCL20 activation

**Fig. 4:**
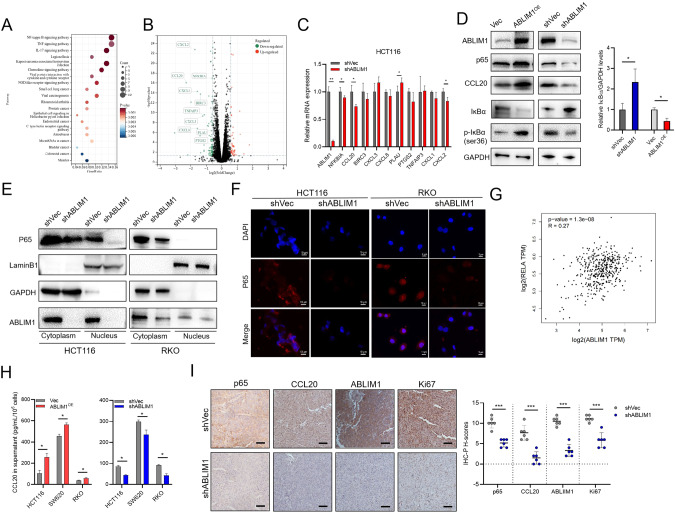
ABLIM1 modulates IκBα/NF-κB/CCL20 activation. **A** The top 20 enriched KEGG pathways for differentially expressed genes (DEGs) between shABLIM1- and shVec-infected HCT116 cells. **B** The volcano plot displayed the up-regulated and down-regulated DEGs between shABLIM1- and shVec-infected HCT116 cells. The top 10 DEGs enriched with NF-κB signaling were labeled. **C** RT-QPCR results of ABLIM1 and the 10 DEGs. **D** Western blot images for ABLIM1, p65, IκBα, p-IκBα (ser36), CCL20, and GAPDH in ABLIM1- overexpressed or silenced HCT 116 cells. Relative IκBα/GAPDH protein levels were quantificated using 3 independent immunoblots. **E** Effect of ABLIM1 knock-down on cytoplasmic and nucleus p65 expressions in HCT116 and RKO cells. **F** Immunofluorescence images stained with anti-P65 antibody and DAPI showed ABLIM1 silence impaired p65 expression intensities in HCT116 and RKO cells. **G** Correlation analysis of ABLIM1 and RELA transcriptional levels in colorectal tumors and adjacent normal samples from the GEPIA2 database using Pearson method. *P* = 1.3e-8, *R* = 0.27. **H** Secreted CCL20 levels in culture supernatants of HCT116, RKO, and SW620 cells determined by ELISA kit. *n* = 3 for each group. **I** Representative p65, Ki-67, ABLIM1, and CCL20 immunohistochemical images of CRC xenografts from nude mice and the semiquantitative analysis upon H-score method. *n* = 5 for each group. For *n* > 3, paired student’s *t*-test; For *n* = 3, Kruskal–Wallis test. ^*^P < 0.05^; **^P < 0.01; ^***^P < 0.001.

Next, we explored the detailed molecular mechanism underlying ABLIM1’s oncogenic role. Stable shABLIM1 and negative control HCT116 cells were subjected to transcriptomic sequencing. Ninety-eight differentially expressed genes (DEGs) were down-regulated and 50 DEGs were up-regulated (Supplementary Table S1). KEGG pathway enrichment was conducted for the DEGs and the top 20 pathways upon the *P* values were listed in Fig. 4A. Of special interest, NF-κB signaling obtained the most significant *P* value and meanwhile the highest gene ratios (14.93%). We visualized the top 10 genes associated with NF-κB signaling in the volcano plot based on the foldchange, including CCL20, CXCL2, CXCL8, CXCL1, BIRC3, PLAU, PTGS2, CXCL3, TNFAIP3, and NFKBIA, all of which were repressed in RNA-seq after ABLIM1 depletion (Fig. 4B). We further testified the mRNA expressions of these 10 genes and found CCL20, CXCL2, NFKBIA, and PLAU were significantly changed after ABLIM1 depletion (Fig. 4C). Since the enriched NF-κB signaling was dramatically down-regulated and NF-κB/CCL20 axis was previously reported to mediate CRC progression [24, 25], we hypothesized that ABLIM1 accelerates CRC growth and metastasis through modulating NF-κB/CCL20 activation. To testify the hypothesis, the protein changes of p65, IĸBα, p-IĸBα, and CCL20 after ABLIM1 overexpression and silence were analyzed, which revealed ABLIM1 could promote NF-κB activation and CCL20 production via modulating IĸBα (Fig. 4D). Nuclear and cytoplasmic protein examination unveiled that ABLIM1 depletion repressed cytoplasmic p65 and its nuclear translocation, which was further confirmed by inhibited p65 expression intensities in both cytoplasm and nucleus in IF images (Fig. 4E, F). Additionally, mRNA expression levels of *RELA* in colorectal cancers and normal colorectal tissues from the GEPIA2 database were positively correlated with those of ABLIM1 (Fig. 4G), supporting the regulation of ABLIM1 on p65. In consideration that NF-κB/CCL20 axis could mediate CRC progression, we analyzed the CCL20 secretion after ABLIM1 manipulation. As would be expected, ABLIM1 overexpression improved the CCL20 production while ABLIM1 silence impaired the CCL20 secretion (Fig. 4H). Consistently, the p65, CCL20, and Ki-67 expressions in tumor xenografts were repressed after ABLIM1 knock-down (Fig. 4I), supporting ABLIM1 modulates the IĸBα/NF-κB/CCL20 axis to promote CRC progression.

### IĸBα/NF-κB p65 intervention reverses the tumor phenotypes induced by ABLIM1 alteration

**Fig. 5:**
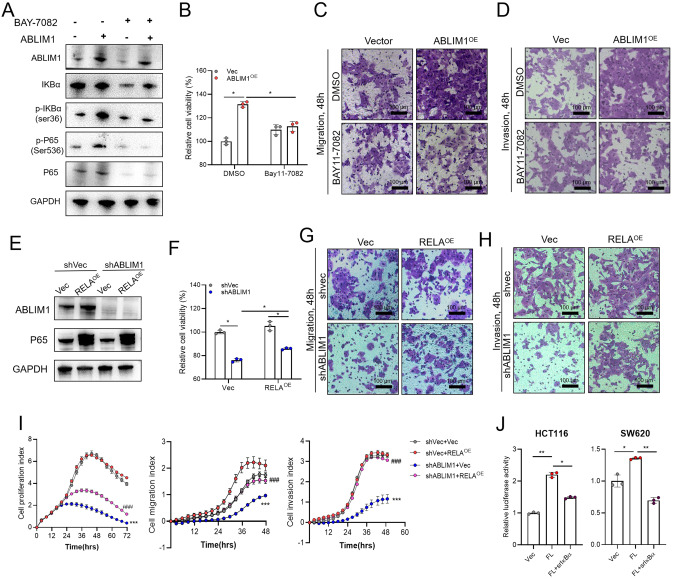
IκBα/NF-κB p65 intervention reversed the phenotypes induced by ABLIM1 alteration. **A** Western blots for p65, p-P65, IκBα, p-IκBα, and ABLIM1 in HCT116 cells overexpressed with ABLIM1 or empty vector. Cells were treated with BAY11-7082 (10 μM) or DMSO control for 8 h before harvest. **B** Effect of BAY11-7082 treatment (10 μM, 24 h) on cell viabilities of HCT116 cells overexpressed with ABLIM1 or empty vector was detected by CCK-8 kit. *n* = 3 for each group. Kruskal–Wallis test. Influence of BAY11-7082 treatment on cell migration **C** and invasion **D** capabilities of HCT116 cells. **E** Western blots of P65 and ABLIM1 for validation of RELA overexpression efficiency in stable shABLIM1 or shVec HCT116 cells. **F** Cell viabilities assayed by CCK-8 kit revealed the effects of RELA overexpression on proliferation of stable shABLIM1 or shVec HCT116 cells. *n* = 3 for each group. Kruskal–Wallis test. Effect of RELA overexpression on HCT116 cell migration **G** and invasion **H** in Transwells. **I** RTCA monitored the influence of RELA overexpression on CRC cell proliferation, migration, and invasion abilities in HCT116 cells with or without ABLIM1 knockdown. *n* = 4 for each group. ^***^P < 0.001, indicated the statistical significances between shVec+Vec group and shABLIM1+Vec group at the end of assays. ^###^*P* < 0.01, indicated the statistical significances between shABLIM1+RELA^OE^ group and shABLIM1+Vec group at the end of assays. Paired *t* test. **J** 48 h after transfection of IκBα S32/36 A phosphorylation mutant, full length ABLIM1, or both, NF-κB transcriptional activities in HCT116 and SW620 cells were detected by dual-luciferase assay. *n* = 3 for each group. Kruskal–Wallis test.

Given that IĸBα/NF-κB p65 mediates the oncogenic role of ABLIM1 in CRC, we testified the influence of IĸBα or NF-κB p65 rescue on the cellular phenotypes induced by ABLIM1 alteration. BAY11-7082, a selective inhibitor of IĸBα phosphorylation [26], was used to treat CRC cells overexpressed with ABLIM1. Immunoblotting images illustrated that BAY11-7082 reversed the IĸBα phosphorylation induced by ABLIM1 up-regulation and thus counteracted the activation effect on NF-κB p65 (Fig. 5A). Importantly, the IĸBα/NF-κB blockade by BAY11-7082 further abolished the effect of ABLIM1 overexpression on cell proliferation, migration, and invasion (Fig. 5B–D), implying IĸBα phosphorylation mediated NF-κB p65 activation is involved in the tumor-promoting role of ABLIM1. Moreover, we investigated whether p65 overexpression could rescue the effect of ABLIM1 knock-down on cell phenotypes. The successful p65 transduction in HCT116 stable cells was confirmed using immunoblotting (Fig. 5E). As displayed in Fig. 5F, *RELA* transduction attenuated the repressive effect of ABLIM1 silence on cell proliferation in CCK-8 assay. Crystal violet stained images of migrated and invaded cells in Transwells also unveiled that *RELA* overexpression could weaken the suppressive influence of ABLIM1 depletion on cell migration and invasion (Fig. 5G, H). In line with these results, the rescue effect of *RELA* overexpression on cell proliferation, migration, and invasion of CRC cells was further validated in RTCA system (Fig. 5I). We also introduced the non-phosphorylation mutant (S32/36 A) of IĸBα (srIĸBα) to determine whether ABLIM1 activates p65 signaling through IĸBα. This state of IĸBα cannot be phosphorylated and prevents the degradation of IĸBα [10]. Dual-luciferase assay showed the presence of srIĸBα diminished the augmented NF-κB transcriptional activity caused by ABLIM1 overexpression in both HCT116 and SW620 cells (Fig. 5J).

### ABLIM1 interacts with IĸBα and facilitates its ubiquitination

**Fig. 6:**
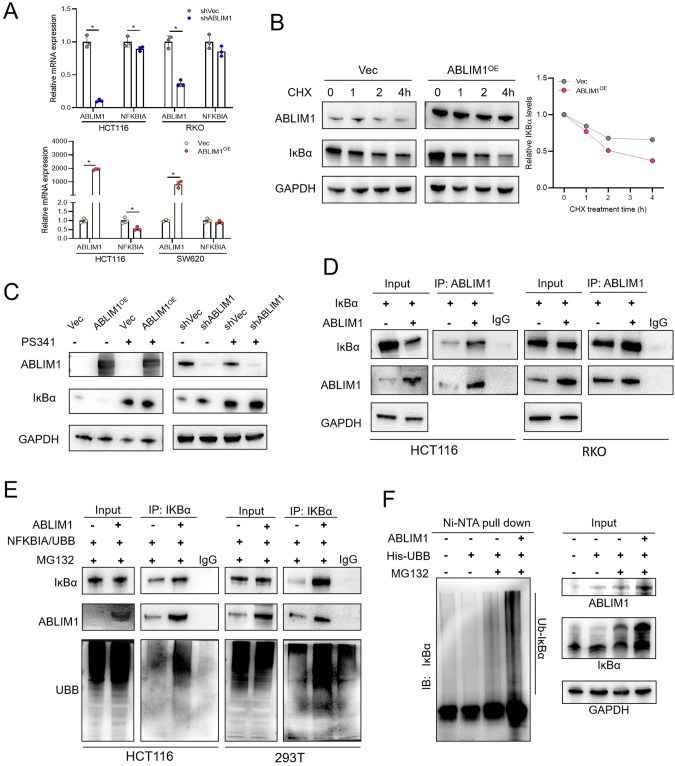
ABLIM1 interacts with IκBα and facilitates its ubiquitination. **A** Relative mRNA levels of NFKBIA and ABLIM1 in HCT116, RKO, and SW620 cells assayed by qRT-PCR. *n* = 3 for each group. Kruskal–Wallis test: ^*^P < 0.05. **B** Immunoblotting of IκBα and ABLIM1 in HCT116 cells treated with CHX (10 μM) for indicated time. Quantification of the IκBα levels relative to GAPDH expression was displayed at the right. **C** Immunoblotting results of IκBα and ABLIM1 in ABLIM1 knockdown or overexpression HCT116 cells treated with PS341 (100 nM). **D** Co-immunoprecipitation assays revealed the interaction between ABLIM1 and IκBα in HCT116 and RKO cells. Cells were transfected with NFKBIA in combination with ABLIM1 or empty vector for 48 h. In control IP, IgG was used instead of ABLIM1 antibody. **E** Co-immunoprecipitation assayed the effect of ABLIM1 overexpression on IκBα ubiquitination in HCT116 and 293 T cells. Cells were transfected with NFKBIA, UBB, and ABLIM1 or empty vector for 48 h and treated with MG132 (20 μM) for 4 h before harvest. **F** In vivo ubiquitination assay using Ni-NTA pull-down confirmed that ABLIM1 promotes the poly-ubiquitination of IκBα.

As above data revealed, ABLIM1 could regulate IĸBα protein levels and phosphorylation. Therefore, we examined whether ABLIM1 could regulate IĸBα mRNA directly. Of special interest, ABLIM1 depletion or overexpression did not influence *NFKBIA* mRNA levels in both SW620 and RKO cells, suggesting ABLIM1 might regulate the level of IĸBα protein largely by affecting its stability. Additionally, ABLIM1 manipulation had a mild impact on transcriptional levels of *NFKBIA* in HCT116 cells (Fig. 6A). Then, cycloheximide (CHX), a protein synthesis inhibitor, was utilized to evaluate the influence of ABLIM1 on the protein stability of IĸBα. As shown in Fig. 6B, the immunoblotting images and its semiquantitative results demonstrated that ABLIM1 overexpression shortened the half-life of endogenous IĸBα. Additionally, CRC cells treated with PS341, a selective proteasome inhibitor, showed a rescued effect on IĸBα protein levels no matter those cells were manipulated with ABLIM1 overexpression or knock-down (Fig. 6C), which implies that the modulation of ABLIM1 on IĸBα degradation should be proteasome-dependent. LIM-domain proteins possess similar structures with RING and PHD domains thus they are thought to have E3 ligase activities, though few of them have been verified [6, 12]. A typical one is SLIM, which was reported to have an E3 ubiquitin ligase activity targeting nuclear p65 [6] and STAT [12]. Hence, we speculated that ABLIM1 may interact with IĸBα and contribute to its ubiquitination and subsequent proteosome-mediated degradation. In order to confirm this speculation, HCT116 and RKO cells were transfected with *NFKBIA* in combination with *ABLIM1*, or empty vectors, respectively. Co-IP assays were then conducted with an anti-ABLIM1 antibody or control IgG. Importantly, the interaction of ABLIM1 and IĸBα was detected in both HCT116 and RKO cells (Fig. 6D). Moreover, ABLIM1 and IĸBα could be co-immunoprecipitated despite the introduction of BAY11-7082 or srIĸBα, suggesting phosphorylation does not modulate the interaction between ABLIM1 and IĸBα (Fig. S4, A–C). Furthermore, we sought to investigate the influence of ABLIM1 on IĸBα ubiquitination. The *UBB* and *NFKBIA* were co-transfected into HCT116 or 293 T cells combined with ABLIM1 or its empty control. Thereafter, those cells were subjected to ubiquitin-based immunoprecipitation assay using an anti-IĸBα antibody. The results in Fig. 6E showed that ABLIM1 facilitated the poly-ubiquitination of IĸBα in both HCT116 and 293 T cells. In vivo ubiquitination assay by using Ni-NTA pull-down also confirmed that ABLIM1 could enhance the poly-ubiquitination of IĸBα (Fig. 6F). Together, these data indicate ABLIM1 modulates IĸBα/NF-κB p65 activation via interacting with IĸBα and facilitating its ubiquitination.

### ABLIM1 is a novel E3 ligase, which interacts with IκBα via HP domain and ubiquitinates it depending on 402-778aa

**Fig. 7:**
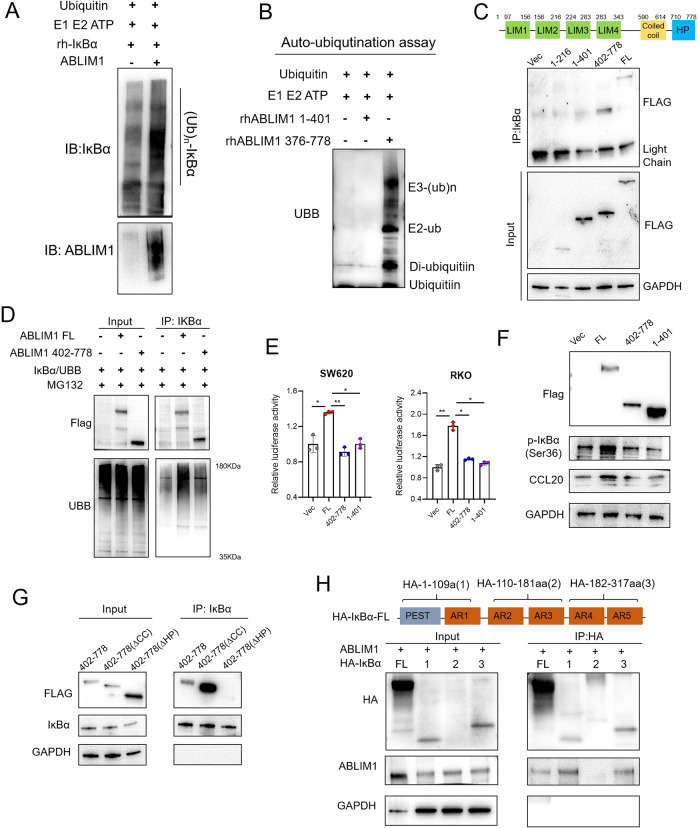
ABLIM1 is a novel E3 ligase, which interacts with IκBα via HP domain and ubiquitinates it depending on 402-778aa. **A** E3 ligase activity of ABLIM1 was determined by in vitro ubiquitination assay. HCT116 cells overexpressed with ABLIM1 or vector underwent IP using ABLIM1 antibody and magnetic beads, and then the beads were incubated with E1, E2, ATP, and recombinant IκBα, followed by immunoblotting examination. **B** Auto-ubiquitination assay revealed that recombinant ABLIM1 376-778aa rather than 1-401aa harbored E3 ligase activity. Ubiquitinated protein was detected by immunoblotting with an anti-ubiquitin antibody. **C** 402-778aa region of ABLIM1 was required for the interaction between ABLIM1 and IκBα. A diagram shows the structures of ABLIM1 (top). HCT116 cells were transfected with Flag-ABLIM1 constructs (full length, 1-216aa, 1-401aa, 402-778aa) or empty vector, respectively. **D** Co-IP assay revealed LIM domains were required for the ubiquitination activity of ABLIM1. **E** NF-κB transcriptional activities in RKO and SW620 cells were detected by dual-luciferase assay after transfection of different ABLIM1 mutant constructs (full length, 1-401aa, 402-778aa). *n* = 3 for each group. Kruskal–Wallis test: ^*^P < 0.05; ^**^P < 0.01^.^ **F** Immunoblotting images of Flag, p-IκBα, CCL20, and GAPDH in HCT116 cells after transfection of different ABLIM1 mutant constructs. **G** In RKO, co-IP assay using ABLIM1 402-778aa mutant constructs (coiled-coil deleted, HP domain deleted) confirmed HP domain was responsible for the interaction between ABLIM1 and IκBα. **H** Western blot images of co-IPs performed on lysates of HCT116 cells transfected with ABLIM1 and indicated HA-IκBα constructs.

To determine whether ABLIM1 possesses E3 ligase activity, in vitro and auto-ubiquitination assays were conducted. Figure 7A indicated ABLIM1 aided IĸBα ubiquitination in vitro and Fig. 7B further revealed that 376-778aa of ABLIM1 rather than LIM domains (1-401aa) was capable of undergoing auto-ubiquitination, which implies ABLIM1 is a novel ubiquitin E3 ligase and 376-778aa is required for the catalysis activity. There are 4 LIM, a coiled-coil, and an HP domains in the structure of ABLIM1. To clarify the detailed region through which ABLIM1 mediates the ubiquitination and interaction with IĸBα, we generated different mutants, including Flag-tagged ABLIM1 1-216aa, 1-401aa, 402-778aa, and full-length (1-778aa) plasmids. We transfected them into HCT116 cells respectively and conducted Co-IP. Notably, apart from the full-length ABLIM1, only ABLIM1 402-778aa could interact with IĸBα, suggesting the 402-778aa region rather than LIM domains are required for the binding of ABLIM1 to IĸBα (Fig. 7C). Next, we investigated the influence of LIM-domain-lacking ABLIM1 mutant (ABLIM1-∆LIM) and full-length ABLIM1, on the poly-ubiquitination of IĸBα and the NF-κB/CCL20 activation. Interestingly, ABLIM1-∆LIM construct (402-778aa) did not enhance the poly-ubiquitination of IĸBα as full-length ABLIM1 did (Fig. 7D), indicating the LIM domains may also play a role in helping protein ubiquitination. Consistently, only 1-401aa or 402-778aa transfection could not enhance the transcriptional activity of NF-κB (Fig. 7E), increase IĸBα phosphorylation, nor promote CCL20 expression (Fig. 7F) when compared with full-length ABLIM1. A possible explanation for this case may be that LIM domains help maintain a favorable 3D structure for ABLIM1 402-778aa to interact and ubiquitinate IĸBα, though they are not essential for the E3 ligase catalysis activity. Actually, proteins containing LIM domains may constitute a new family of ubiquitin E3 ligases, and LIM domains have been reported to be essential for the E3 ligase activity of LIM-domain proteins like SLIM [12]. Next, we explored the functional domain that mediated the interaction between ABLIM1 and IĸBα, a series of deletion mutant plasmids for ABLIM1 (402-778aa∆Coiled-coil, 402-778aa∆HP) and IĸBα (Full-length; 1,1-109aa; 2, 110-181aa; 3, 182-317aa) were transfected and co-IPs were performed in RKO and HCT116. In ABLIM1, HP domain was necessary to bring down IĸBα (Fig. 7G). In IĸBα, PEST, AR1, AR4, and AR5 were required to bring down ABLIM1 (Fig. 7H).

**Fig. 8:**
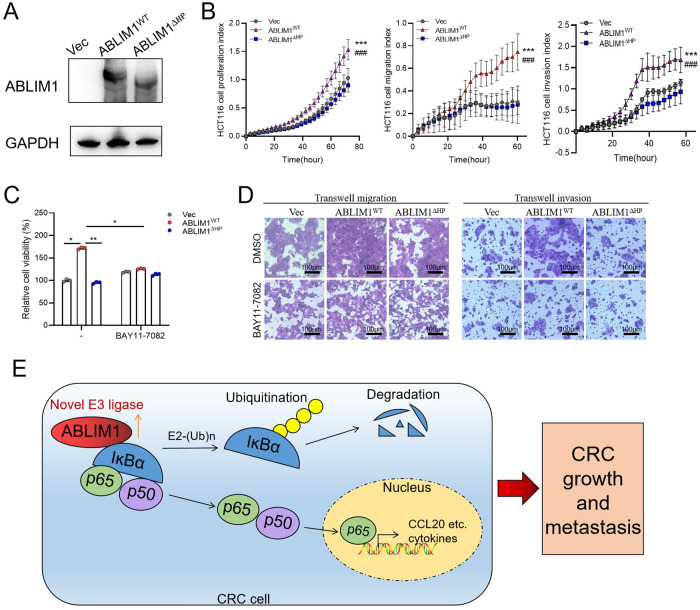
HP domain deletion eliminates the oncogenic role of ABLIM1. **A** Immunoblotting validated the overexpression efficiency of ABLIM1^WT^, ABLIM1^∆HP^, or empty vector in HCT116 cells. **B** HCT116 cell proliferation, migration, and invasion indexes monitored by RTCA system after overexpression with ABLIM1^WT^, ABLIM1^∆HP^, or empty vector. *n* = 4 ~ 6 in each group. Paired *t*-test. ^***^p < 0.001 for ABLIM1^WT^ versus Vec. ^###^*p* < 0.001 for ABLIM1^WT^ versus ABLIM1^∆HP^. **C** HCT116 cell viabilities of indicated groups were assayed by CCK-8 kit after BAY11-7082 treatment. Kruskal–Wallis test: ^*^P < 0.05; ^**^P < 0^.^01. **D** HCT116 cell migration and invasion images stained by crystal violet after indicated treatments. **E** A diagram showing how ABLIM1 mediates the activation of IκBα/NF-κB p65/CCL20 axis and thus augments CRC growth and metastasis.

Given that HP domain was required for both ABLIM1-IĸBα interaction and E3 ligase activity, we observed the rescue effect of HP deletion on CRC cell behaviors. Based on the RTCA data, ABLIM1^∆HP^ overexpression counteracted the promoting role of wild-type ABLIM1 on cell proliferation, migration, and invasion (Fig. 8A, B). Consistently, CCK-8 assay and transwell experiments showed that ABLIM1^∆HP^ group exhibited similar proliferation, migration, and invasion abilities as the empty vector group, while wide type ABLIM1 dramatically enhanced those phenotypes (Fig. 8C, D). Further re-introduction of BAY11-7082 diminished the differences among these 3 groups (Fig. 8C, D). These rescue experiments demonstrate that the HP domain is indispensable for the oncogenic role of ABLIM1.

Taken together, as depicted in Fig. 8E, we clarify that ABLIM1 acts as a novel ubiquitin E3 ligase, which interacts with IĸBα via HP domain, promotes IĸBα ubiquitination, and then activates NF-κB/CCL20 signaling, finally leading to the growth and metastasis in CRC.

## Discussion

Excessive and prolonged activation of NF-κB signaling plays an important role in CRC progression and metastasis. To date, the modulation of NF-κB activity remains not fully understood, since novel E3 ligases targeting IĸBα are continually being identified apart from the SCF complex. In the present study, we unveil that ABLIM1 plays an oncogenic role in CRC growth and metastasis through modulating IĸBα/NF-κB/CCL20 axis. Notably, ABLIM1 is identified as a novel E3 ligase with specificity toward IĸBα for the first time, which interacts with IĸBα via HP domain and ubiquitinates it via distinct catalysis motifs as other LIM-domain proteins. Additionally, highly expressed ABLIM1 in CRC correlated with short disease-free survival time, suggesting ABLIM1 may be a novel prognostic marker in CRC.

ABLIM1 is originally characterized as a cytoskeleton protein binding to actin filaments and is involved in actin-related skeletal rearrangement [22, 27]. Most studies on the function of ABLIM1 focused on its role in regulating actin networks [28, 29], whereas its role and corresponding mechanism in cancers, especially colorectal cancer, remains rarely known. Several studies investigated its low expression profiles in glioblastoma [21] and declared ABLIM1 might act as a tumor suppressor in glioblastoma [21], nasopharyngeal carcinoma [30], and melanoma [22], however the underlying mechanism remains unclear. Here, we found ABLIM1 exhibited a distinct expression profile (highly expressed in CRCs compared with adjacent normal counterparts) in CRC than in glioblastoma and high ABLIM1 expression predicted a short DFS time, indicating a different role of ABLIM1 in CRC. We proved that ABLIM1 overexpression could promote CRC cell growth, migration, and metastasis while ABLIM1 knock-down inhibited these progressive behaviors in vitro. Further tumor xenografts and liver metastatic models validated the oncogenic role of ABLIM1 in vivo. Mechanistically, we elucidated ABLIM1 ubiquitinated IĸBα, promoted NF-κB activation, and thus induced CCL20 expression, causing the growth and metastasis of CRC cells. These findings revealed the diverse functions of ABLIM1 in different cancers, which might not only associate with the heterogeneity across tumors but also correlate with the molecular scaffold property of LIM-domain family proteins [14]. Similarly, diverse functions in cancers have been observed in many LIM-domain proteins, like LMO-only proteins [14] and PDLIM2 [6, 12], which could be ascribed to the diverse interacting partners.

Extensive studies discussed the crucial role of NF-κB/CCL20 axis in tumor growth and metastasis, including CRC metastasis [31–33]. Here, we found CCL20 was the most down-regulated cytokine after ABLIM1 silence in RNA-seq (Table S1), and CCL20 secretion was enhanced after ABLIM1 overexpression, indicating NF-κB-induced CCL20 might be involved in the growth and metastasis of CRC modulated by ABLIM1. Consistently, CCL20 expression has been confirmed to be tightly regulated by NF-κB and of special importance in facilitating tumor growth and metastasis through interacting with CCR6 in CRC [31, 33]. In addition, increased CCL20 could further activate NF-κB signaling and thus form a feedforward loop to drive tumor progression in multiple cancers [32, 34], which needs further validation in CRC.

Of note, we uncovered ABLIM1 as a novel E3 ligase binding to IĸBα. IĸBα ubiquitination is a crucial step for NF-κB p65/p50 dimer release and activation, and SCF complex may be the firstly proven E3 ligase targeting IĸBα ubiquitination [35]. However, subsequent studies proved IĸBα could still be degraded after SCF knock-out, suggesting there are other E3 ligases that could act on ubiquitination and proteasomal-mediated degradation of IĸBα [10]. LIM-domain Zn finger modules harbor RING and PHD-like structures so that they have been predicted to constitute a new family of ubiquitin E3 ligases [6, 12, 15, 36], though most of these proteins have not been verified. PDLIM2 may be the first LIM-domain protein that has been confirmed to possess E3 ligase activity via its LIM domains and nuclear p65 and STAT were both its substrates [6, 12]. Interestingly, in our case, 402-778aa in ABLIM1 rather than the LIM domains (1-401aa) seems to be responsible for the E3 ligase catalysis activity, and LIM domains are still required for the IĸBα ubiquitination (Fig. 7B–G). There are several possible explanations for this in consideration of the molecular scaffold function of LIM domains [37, 38]. For instance, LIM domains in ABLIM1 might interact with other proteins to facilitate its E3 ligase activity or help maintain a favorable 3D structure for 402-778aa to ubiquitinate IĸBα. A similar example is the LIM domain of Hic5 [39] and PDLIM7 [40], which have been clarified to facilitate E3 ligase activity through interacting with catalysis domains of E3 ligase. Another interesting point is, none of typical E3 motifs [41, 42], including HECT (homologous to E6-associated protein C-terminus), U-box, RING, LIM, plant homeodomain (PHD), and RING-Cys-Relay, are contained in 402-778aa of ABLIM1. Since the 3D crystal structure of ABLIM1 remains unknown, further studies are needed to clarify the structure nature of ABLIM1, so as the relationship between ABLIM1 LIM domains and its E3 ligase activity.

In summary, we uncovered the oncogenic role of ABLIM1 in CRC growth and metastasis in vitro and in vivo by activating NF-κB/CCL20 signal. Mechanistically, ABLIM1 acts as a novel ubiquitin E3 ligase targeting IĸBα ubiquitination, among which the LIM domain and HP domain of ABLIM1 play distinct functions. Our findings deepen the understanding of IĸBα/NF-κB signaling modulation in CRC and provide novel insights into the functions of LIM-domain proteins in cancer.

### Supplementary information

Supplementary Material-figures and Original WBs

Supplementary Table S1 DEGs

Supplementary Table S2 primer sequences

## Data Availability

The raw RNA-sequencing data reported in this paper have been deposited in the Genome Sequence Archive (Accession number, HRA005193) that are publicly accessible at https://ngdc.cncb.ac.cn/gsa-human. All datasets analyzed in the study are available from the corresponding authors on reasonable request.
