# Supplementary material for: ABLIM1, a novel ubiquitin E3 ligase, promotes growth and metastasis of colorectal cancer through targeting IĸBα ubiquitination and activating NF-ĸB signaling
Source: Cell Death Differ. 2024 Jan 16;31(2):203–16. doi: 10.1038/s41418-024-01256-y (PMC10850134; doi:10.1038/s41418-024-01256-y)
Supplement: Supplementary file 1 — Supplementary Material-figures and Original WBs [file 41418_2024_1256_MOESM1_ESM.pdf]

Supplementary figures

Figure S1

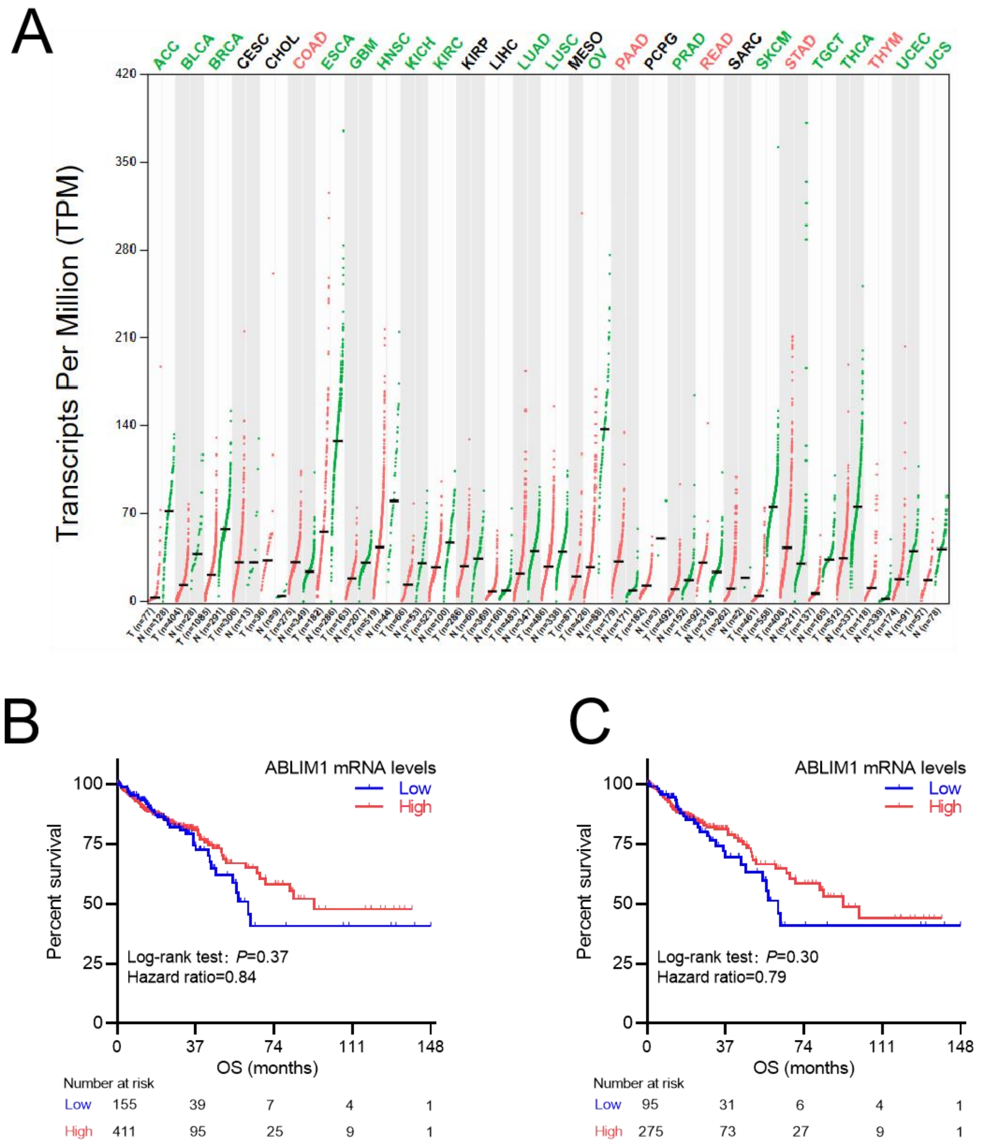

Figure S1. ABLIM1 expression profiles and survival curves. (A) ABLIM1 expression profiles in different tumors analyzed in GEPIA 2 database (http://gepia2.cancer-pku.cn/). Tumors marked in red or blue indicates the gene is significantly up-regulated or down-regulated in tumors. (B) CRC patient overall survival curves stratified by ABLIM1 expression levels using data downloaded from CBioPortal database (B, TCGA PanCancer Atlas; C, TCGA Firehose Legacy). B, P = 0.37, hazard ratio = 0.84 [0.56–1.26, 95% confidence interval (CI)]. C, P = 0.30, hazard ratio = 0.79 [0.49–1.27, 95% CI].

Figure S2

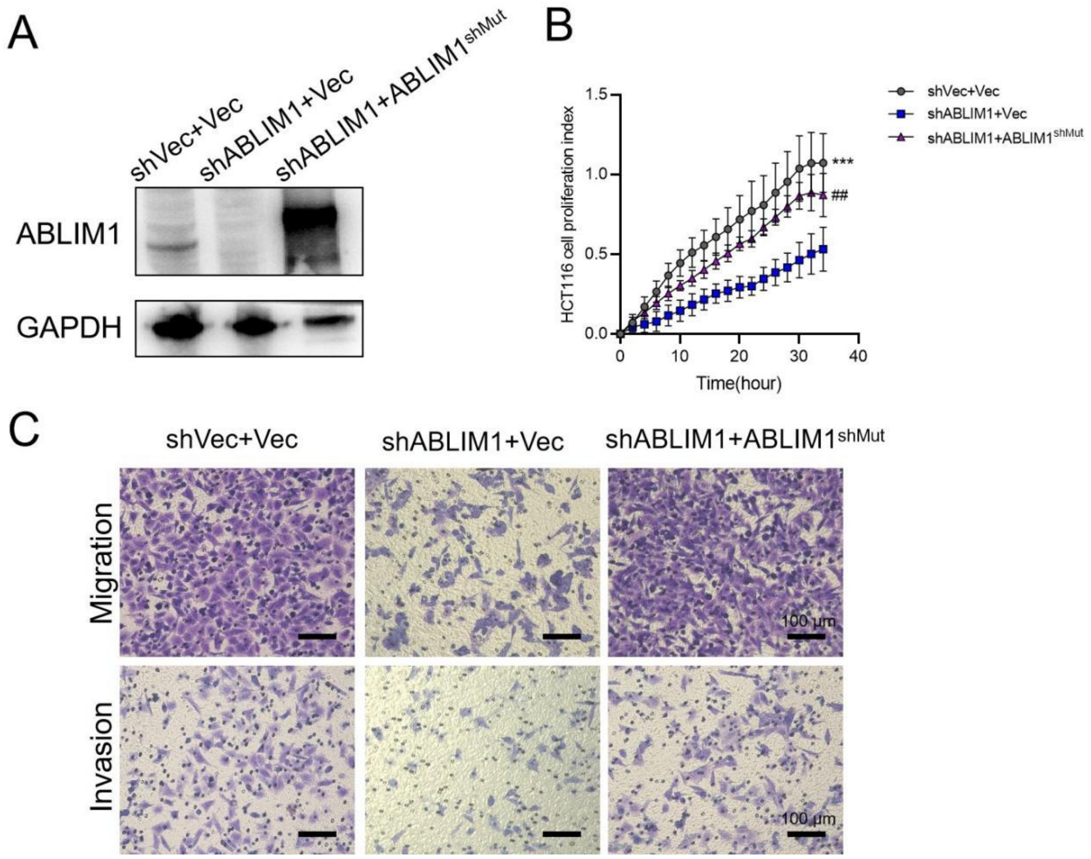

Figure S2. The rescue effect of a shRNA-resistant mutant of ABLIM1 on colorectal cancer cell proliferation, migration, and invasion. (A) Western blot analysis of ABLIM1 in HCT116 cells transduced with shABLIM1+ABLIM1^{shMut}, shABLIM1+Vec, or shVec+Vec. (B) HCT116 cell proliferation indexes recorded by RTCA system after indicated treatments. N = 4 ~ 5 for each group. Unpaired t-test. ***, $p < 0.001$ for shVec+Vec versus shABLIM1+Vec. $^{\#\#}$, $p < 0.01$ for shABLIM1+ABLIM1^{shMut} versus shABLIM1+Vec. (C) HCT116 cell migration and invasion images stained by crystal violet after indicated treatments.

Figure S3

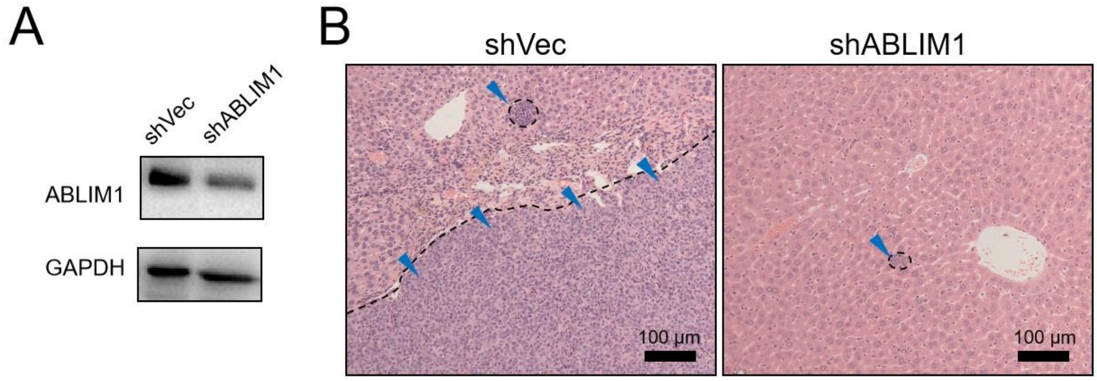

Figure S3. (A) Western blot analysis of ABLIM1 knock-down efficiency in SW620 cells transduced by shABLIM1 or shVec. (B) HE images of nude mouse livers in the CRC liver metastasis models. Stable shVec or shABLIM1 SW620 cells were injected into hepatic portal veins of nude mice (N = 5) to establish the CRC liver metastasis model. Blue triangles indicate the tumor nodes in livers under microscopy.

Figure S4

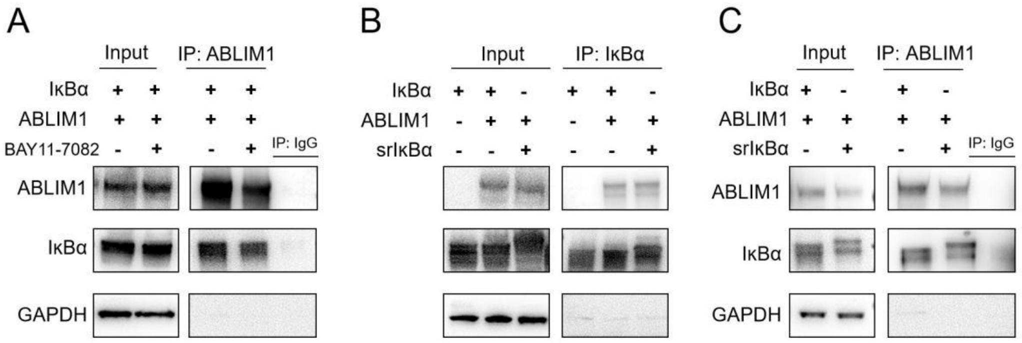

Figure S4. Phosphorylation does not modulate the interaction between ABLIM1 and IκBα. Co-immunoprecipitation assays were performed after BAY11-7082 treatment (10 μM, 16 h) (A) or srIκBα overexpression (B) in 293T cells. (C) In HCT116 cells, ABLIM1 antibody was used to co-immunoprecipitate IκBα after ABLIM1, and srIκBα or IκBα overexpression. ABLIM1, IκBα, and GAPDH were detected by immunoblotting.

Original Western blot images:

Figure 1F:

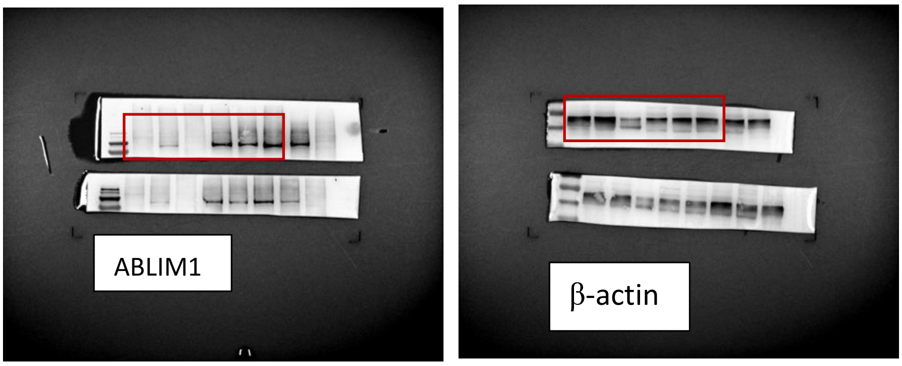

Figure 2A:

HCT116 (UP) and RKO (Down)

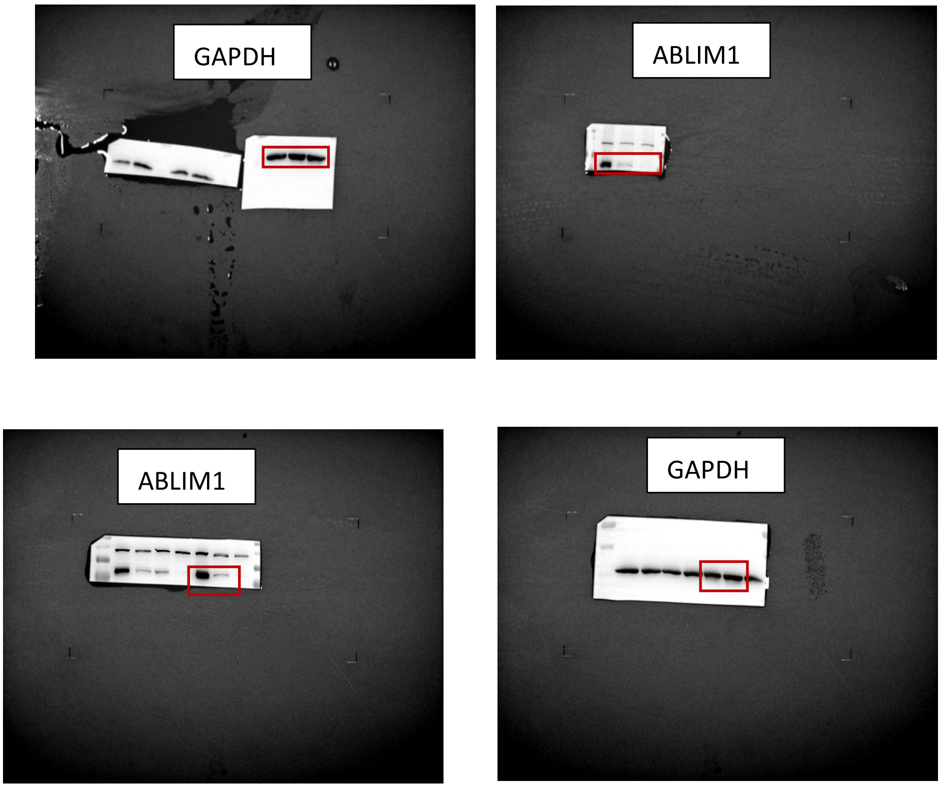

Figure 3A:

HCT116 (Up) and SW620 (Down)

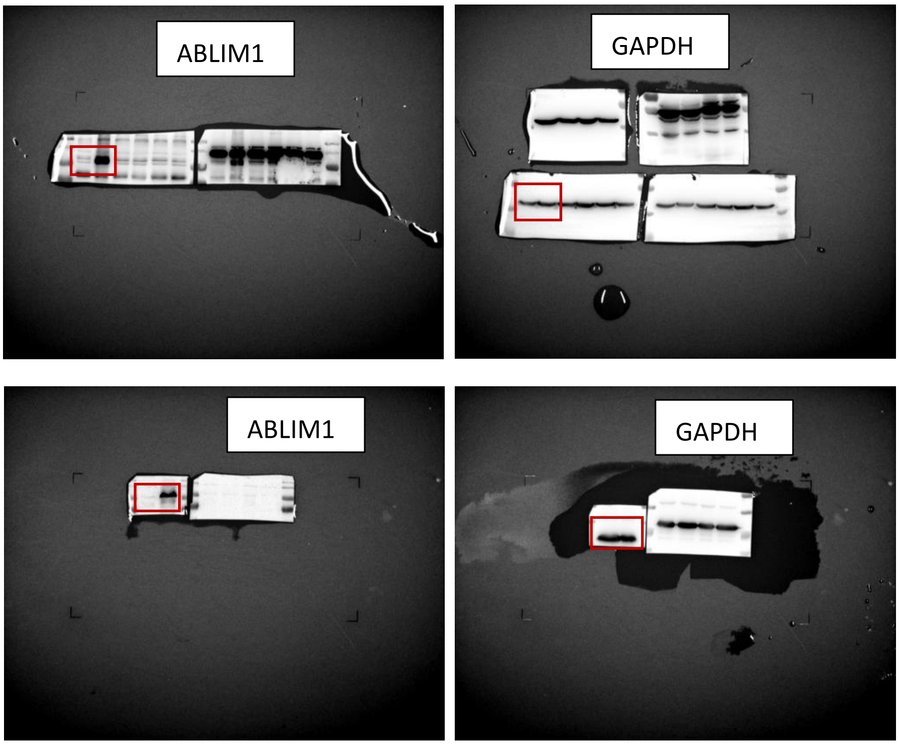

Figure 4D

Vector AND ABLIM1 ^{OE}

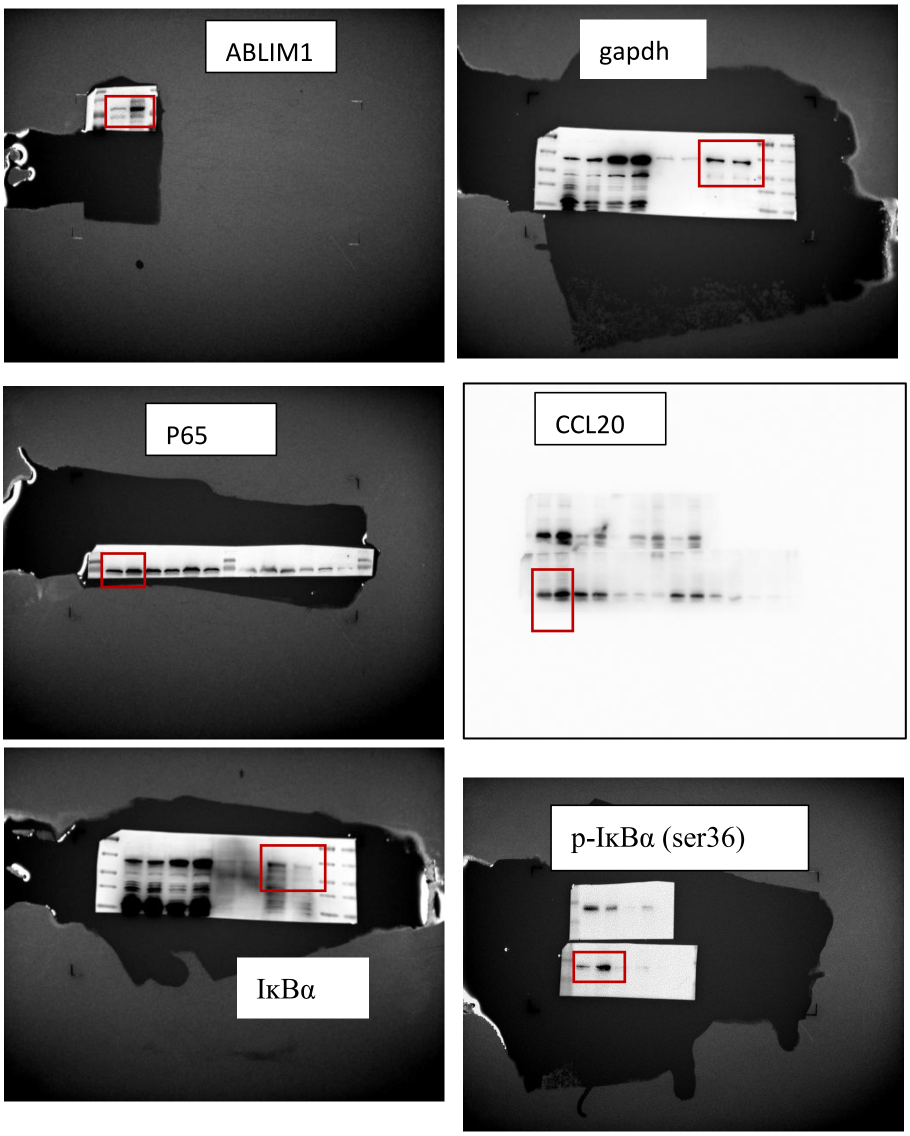

shVec AND shABLIM1

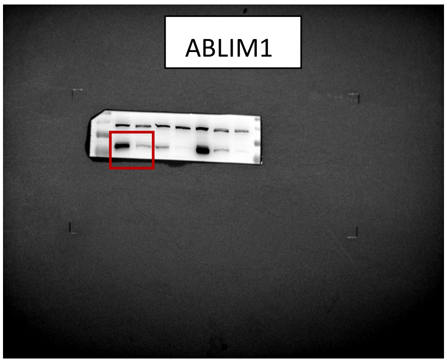

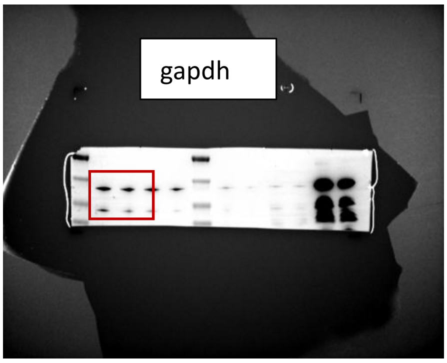

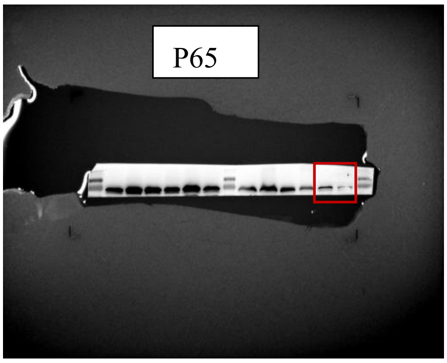

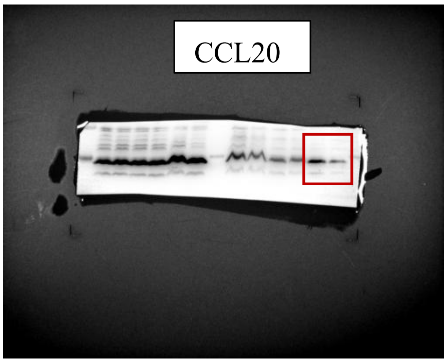

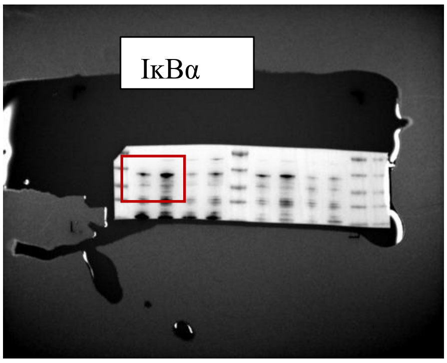

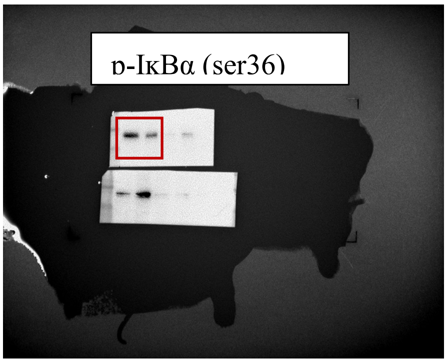

Figure 4E

HCT116:

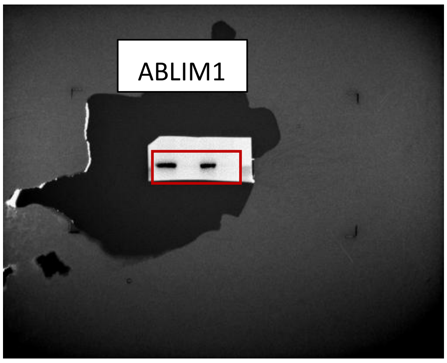

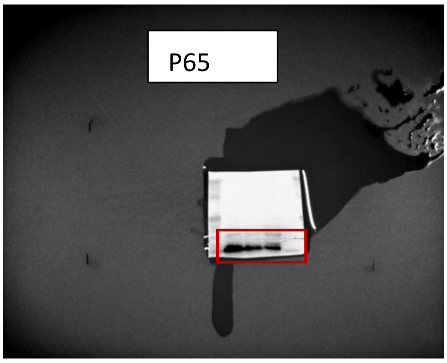

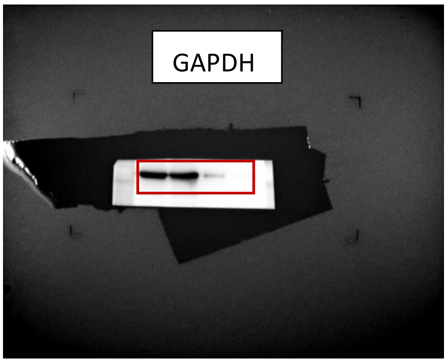

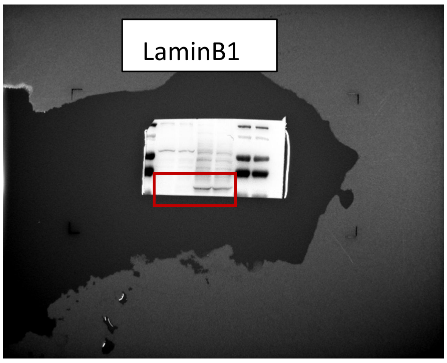

RKO:

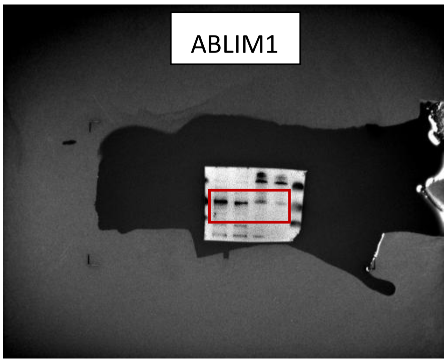

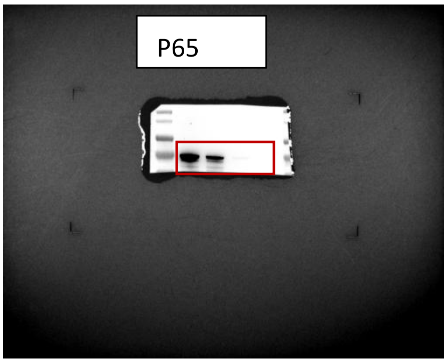

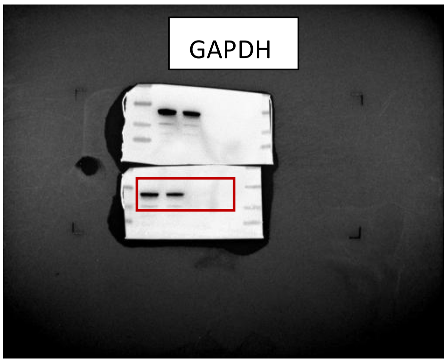

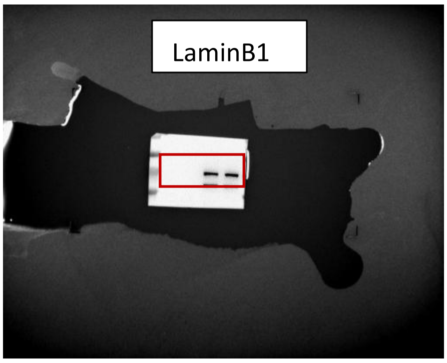

Figure 5A

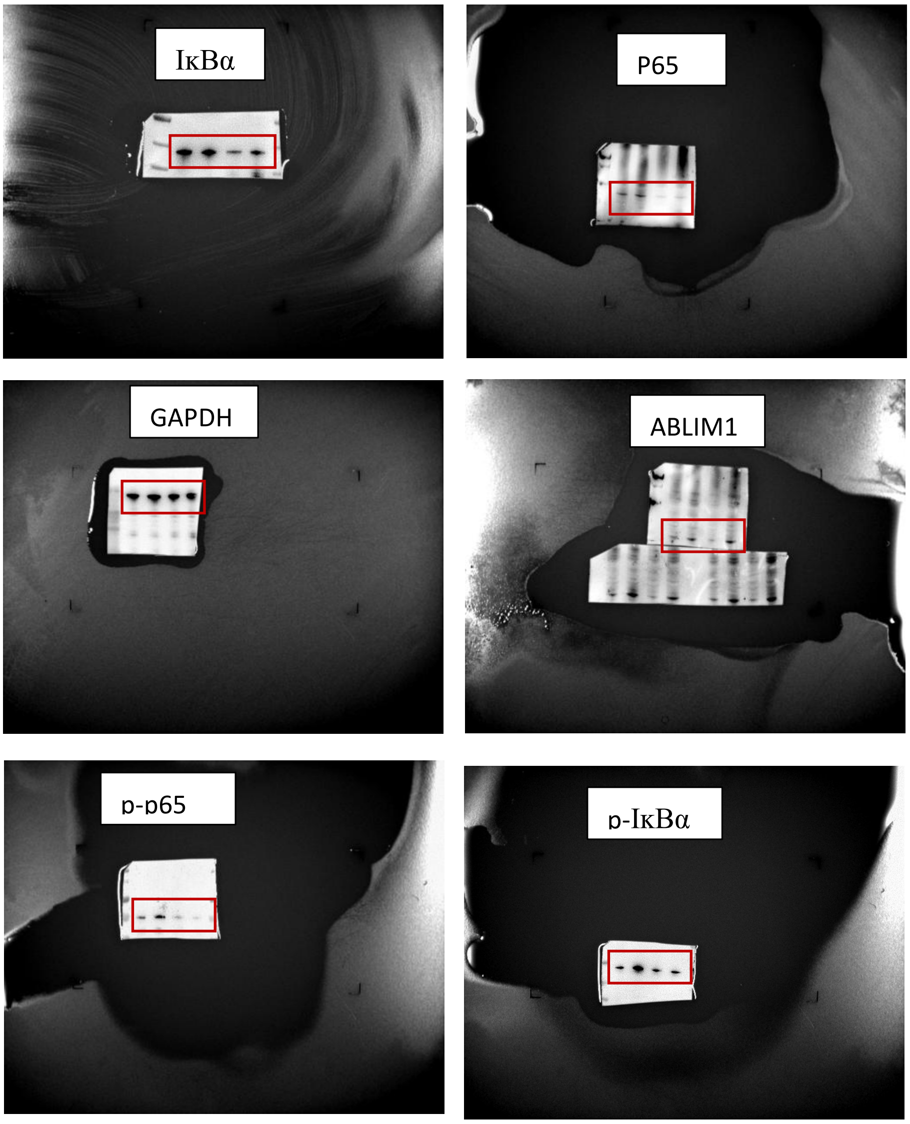

Figure 5E

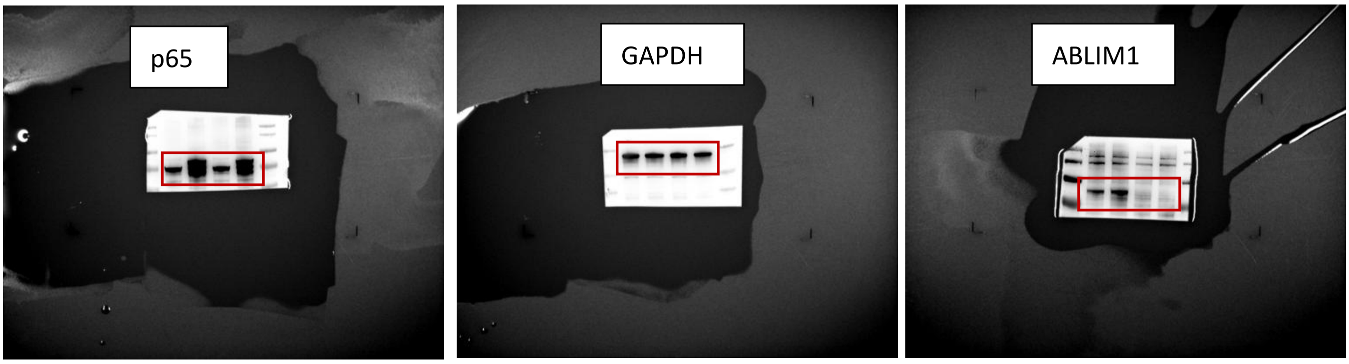

Figure 6B

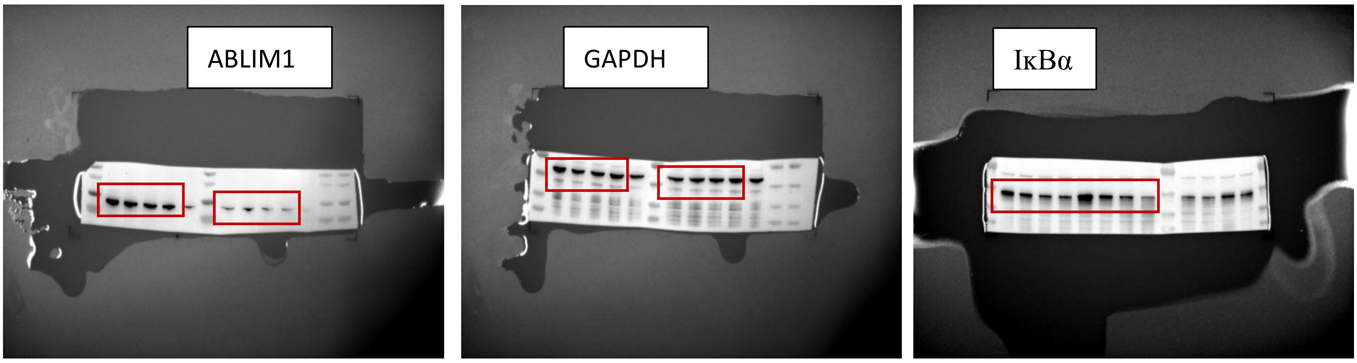

Figure 6C

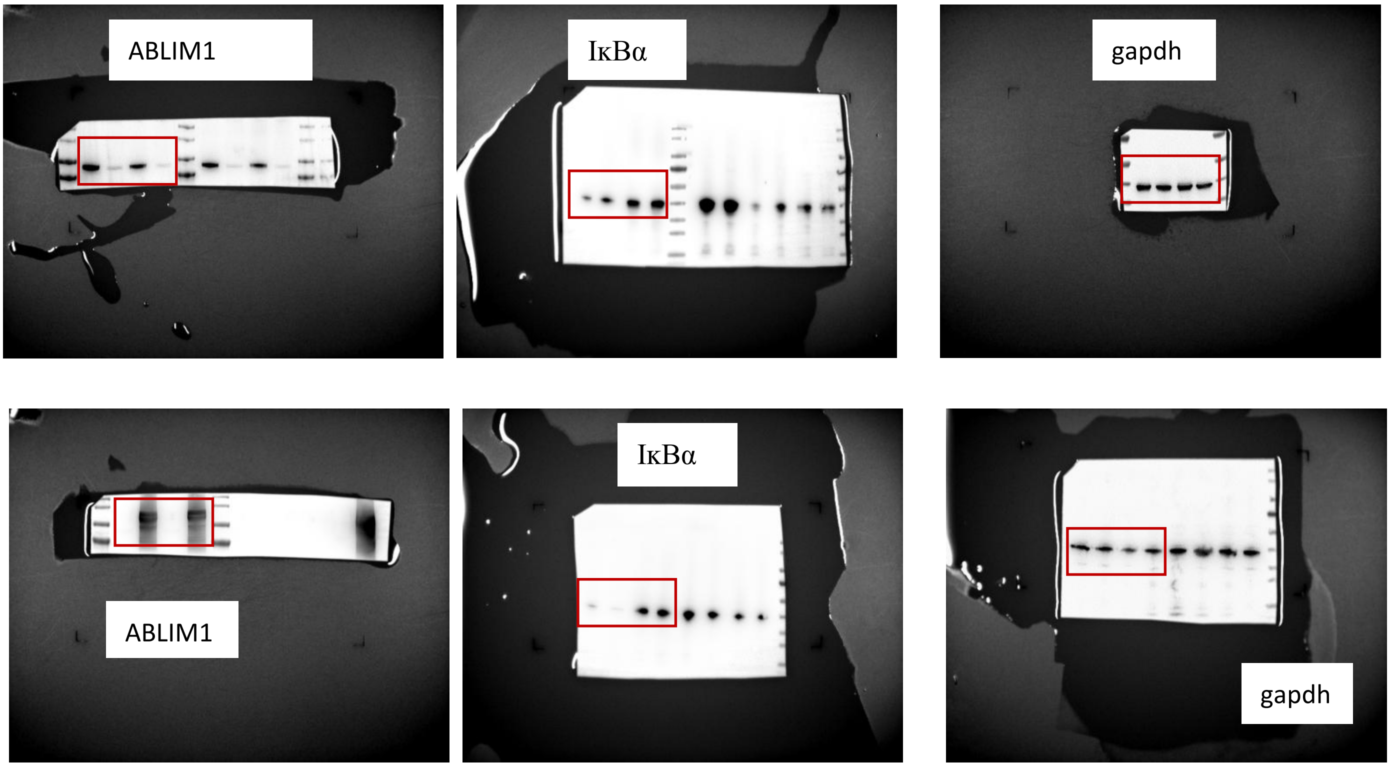

Figure 6D

HCT116

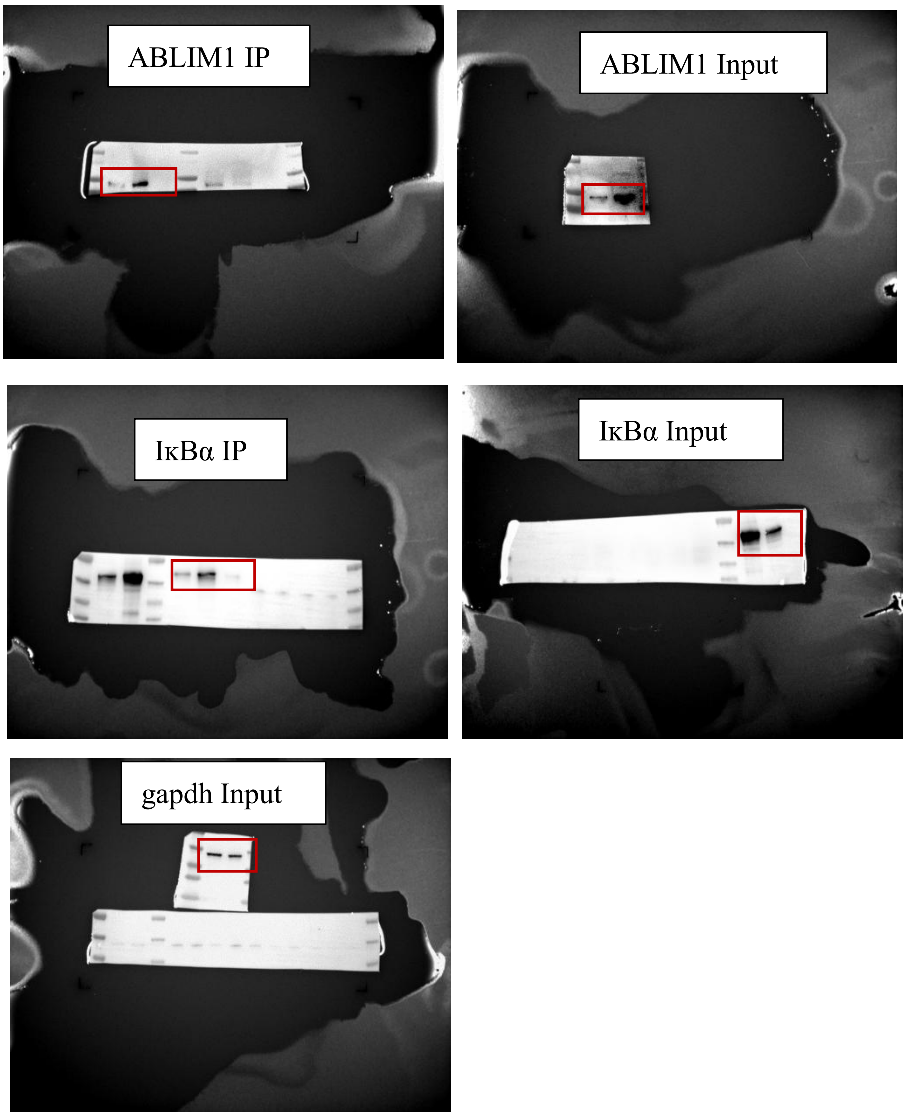

Figure 6D

RKO

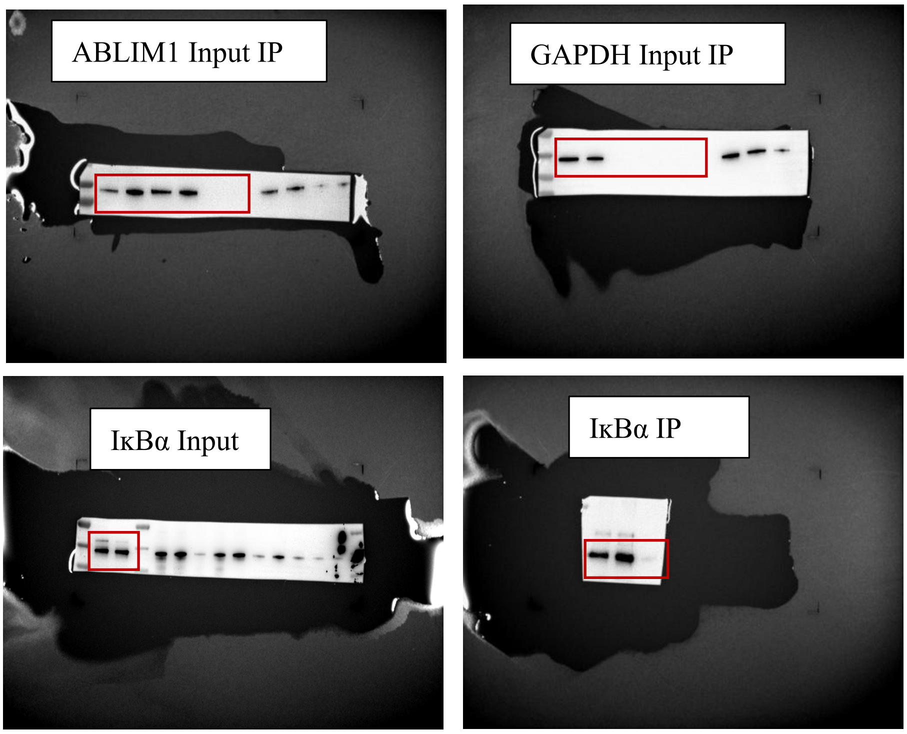

Figure 6E

HCT116

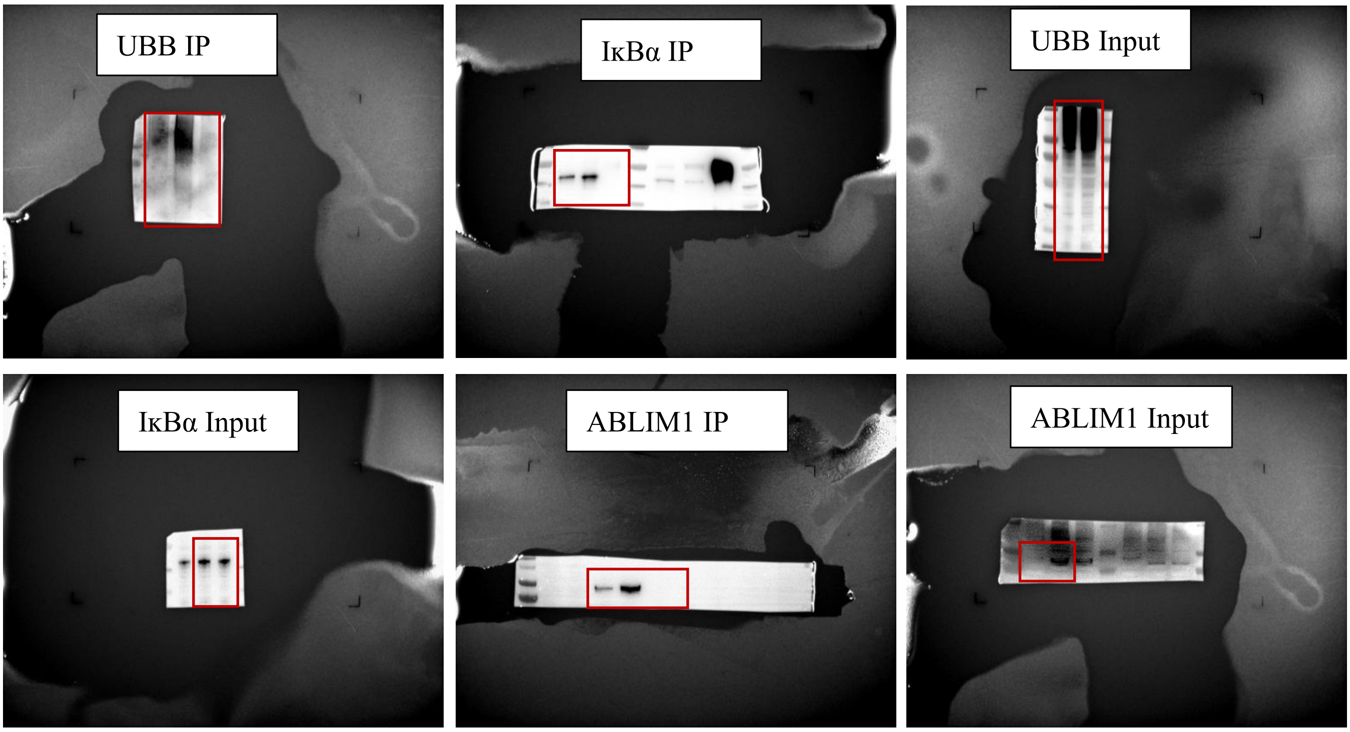

293T:

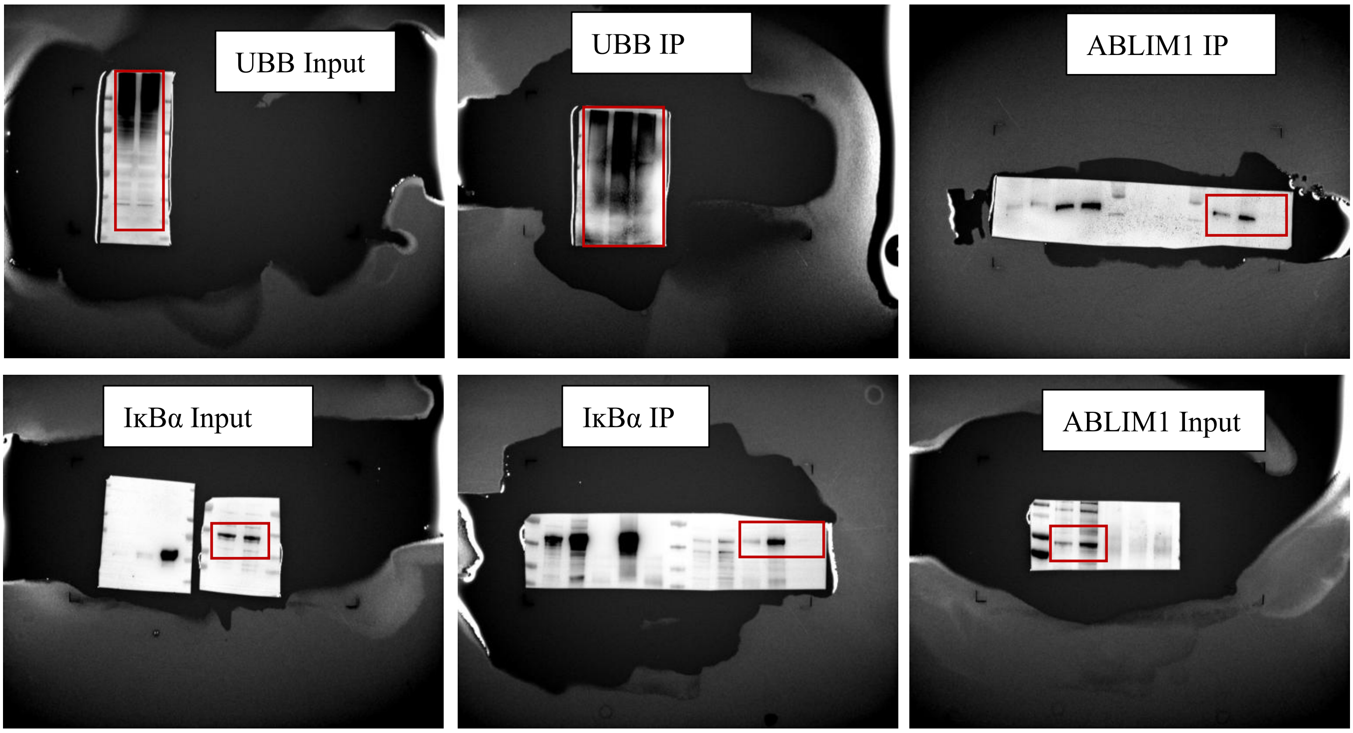

Figure 6F

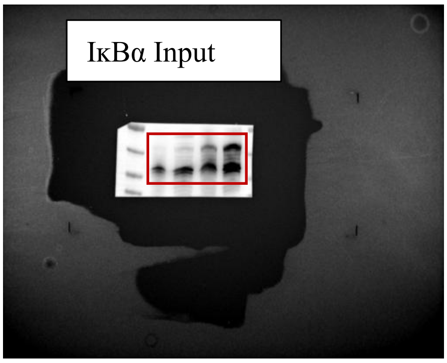

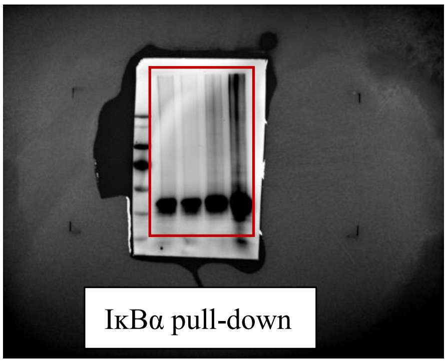

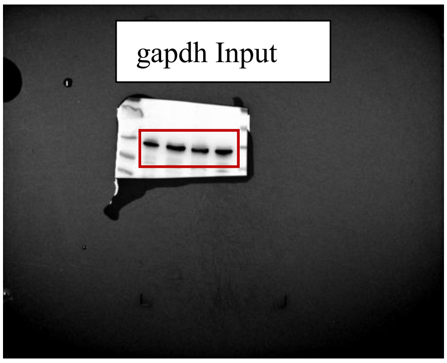

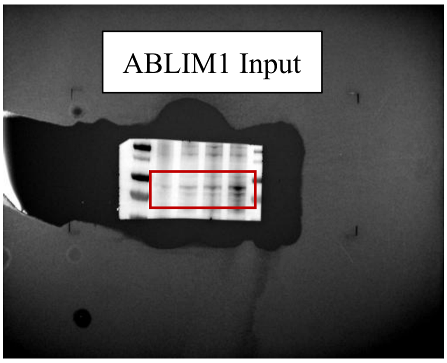

Figure 7A

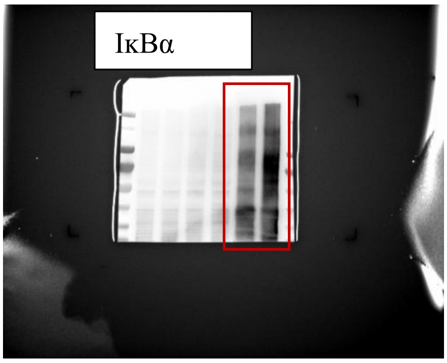

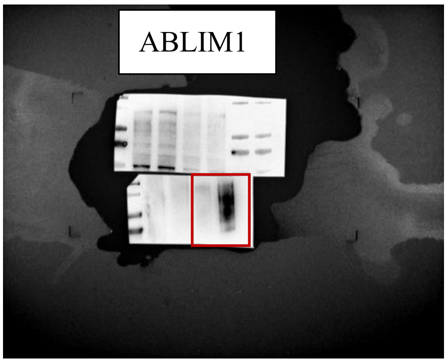

Figure 7B

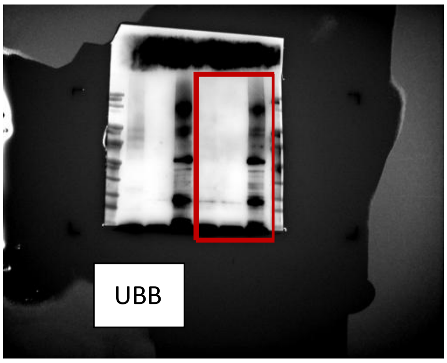

Figure 7C

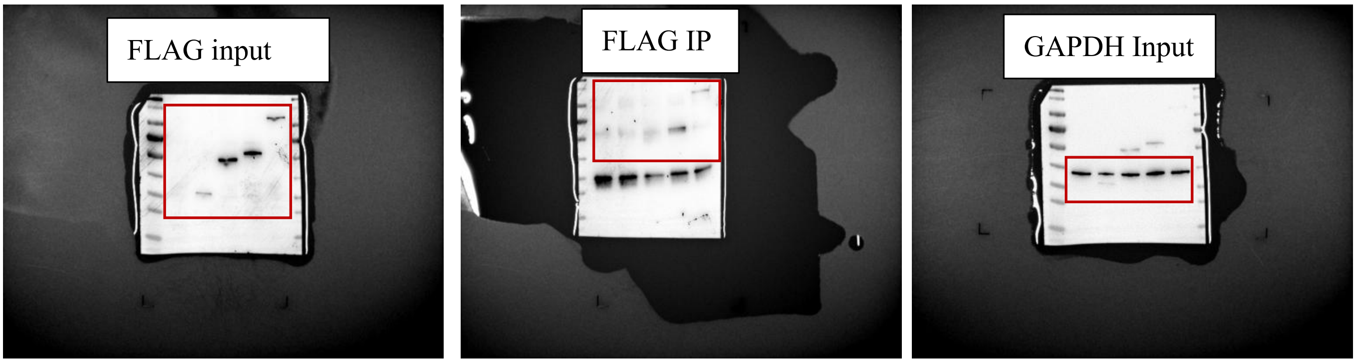

Figure 7D

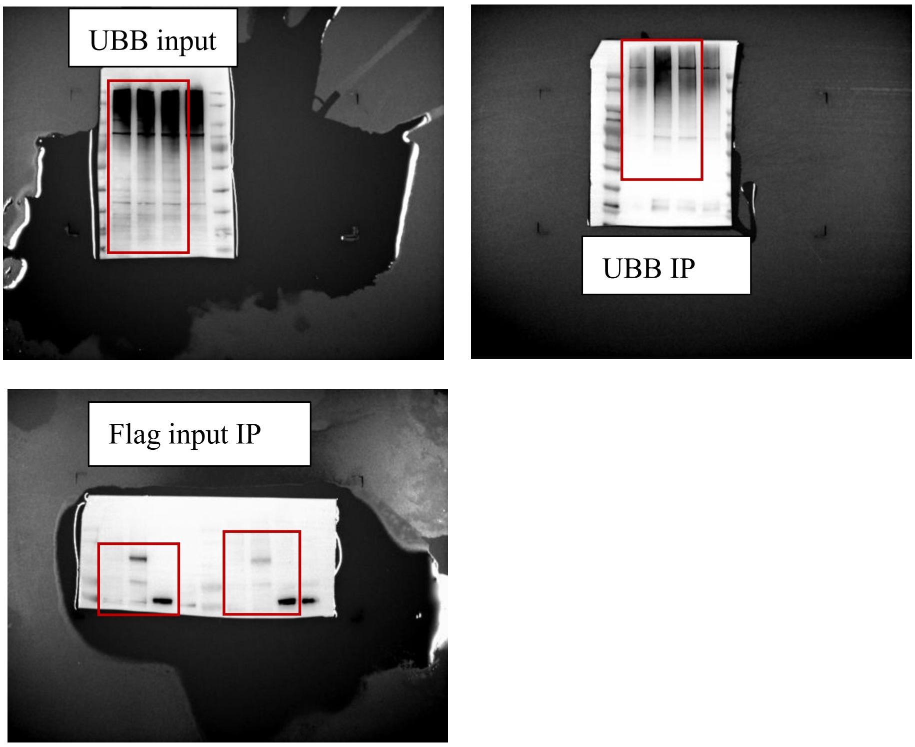

Figure 7F:

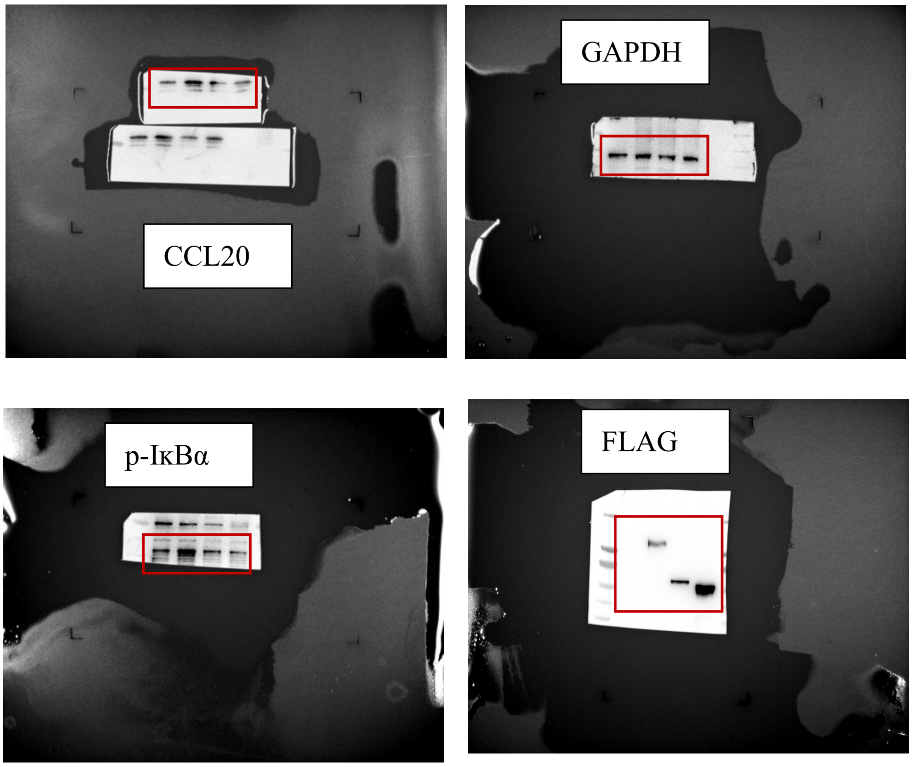

Figure 7G

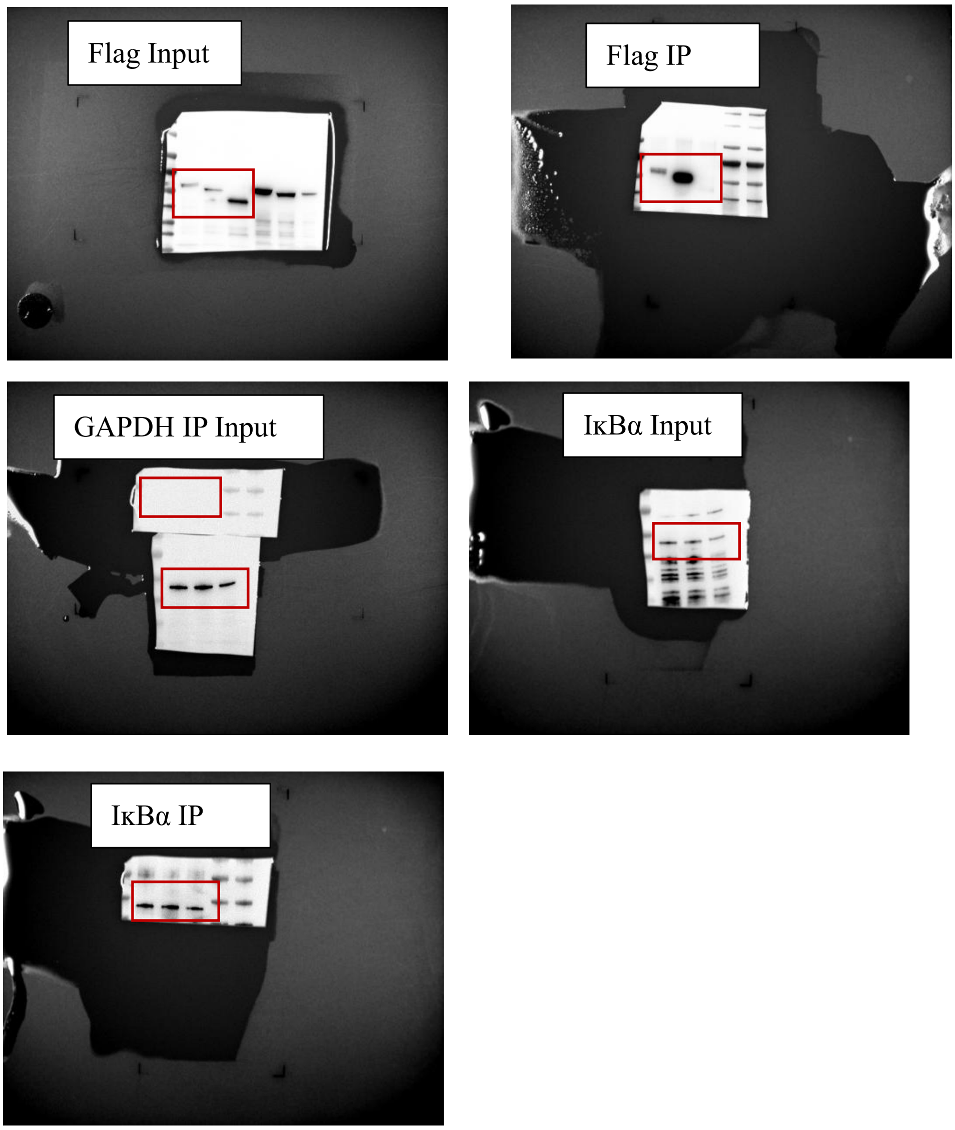

Figure 7H

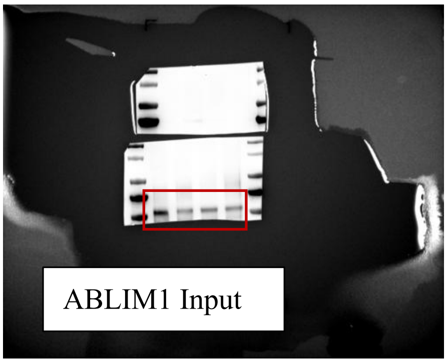

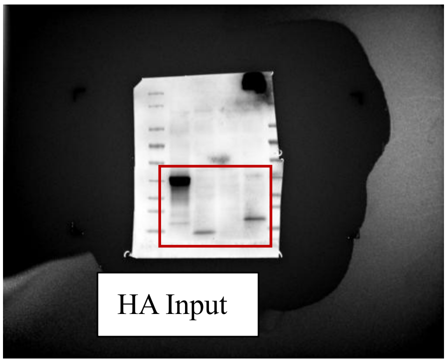

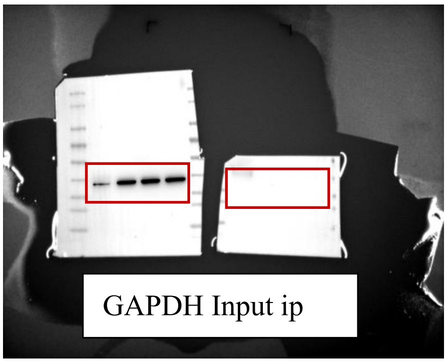

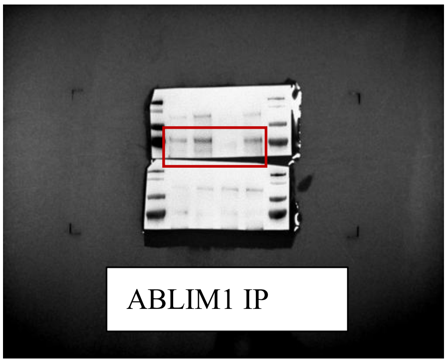

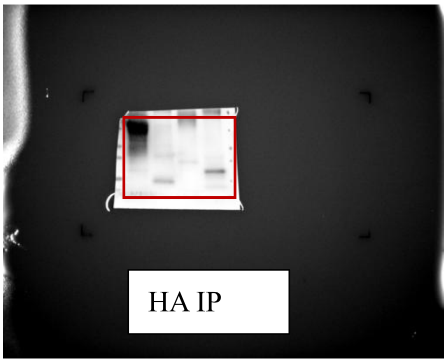

Figure 8A

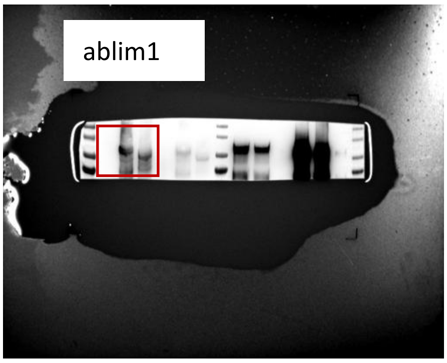

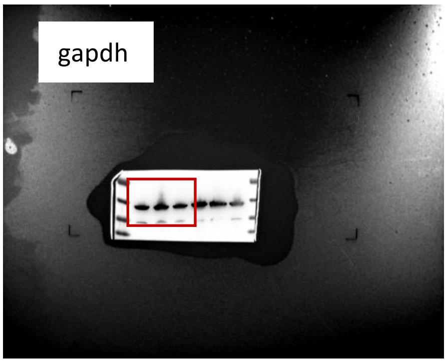

Supplementary Figure S2A

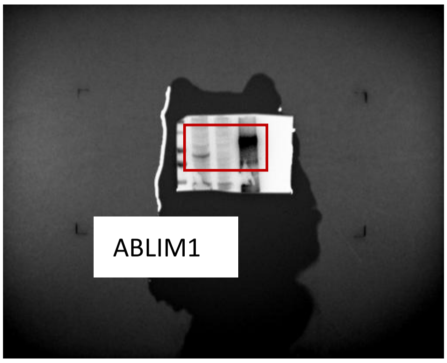

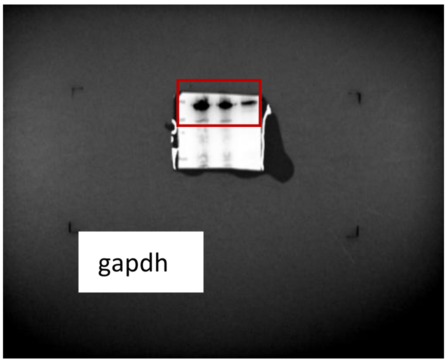

Figure S3A

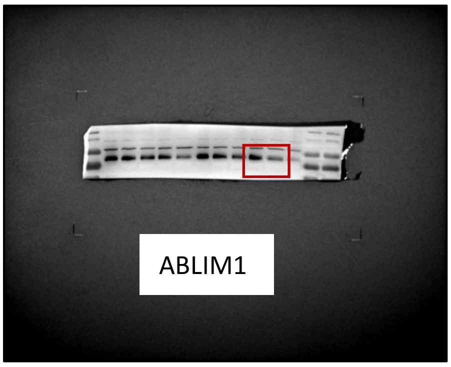

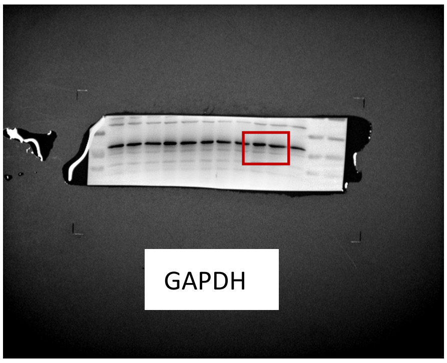

Figure S4A

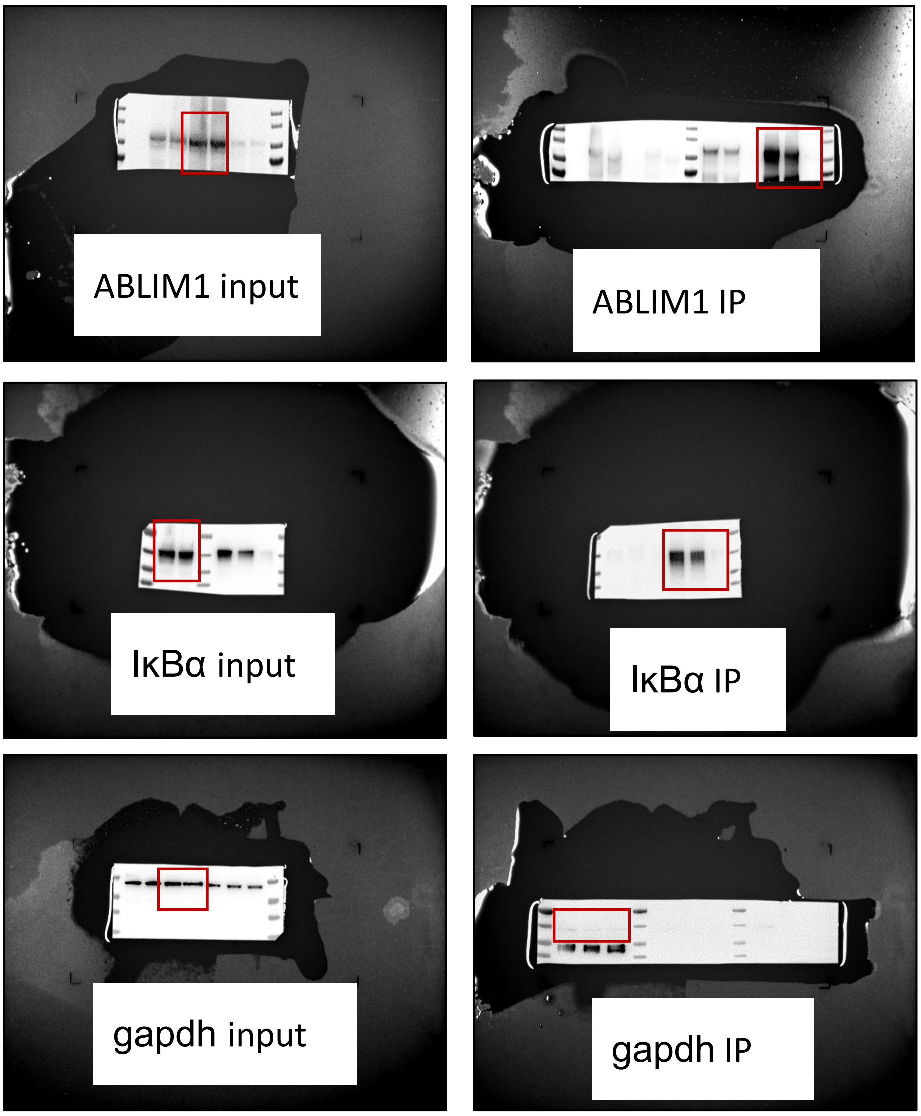
ABLIM1 input
ABLIM1 IP
IκBα input
IκBα IP
gapdh input
gapdh IP

Figure S4B

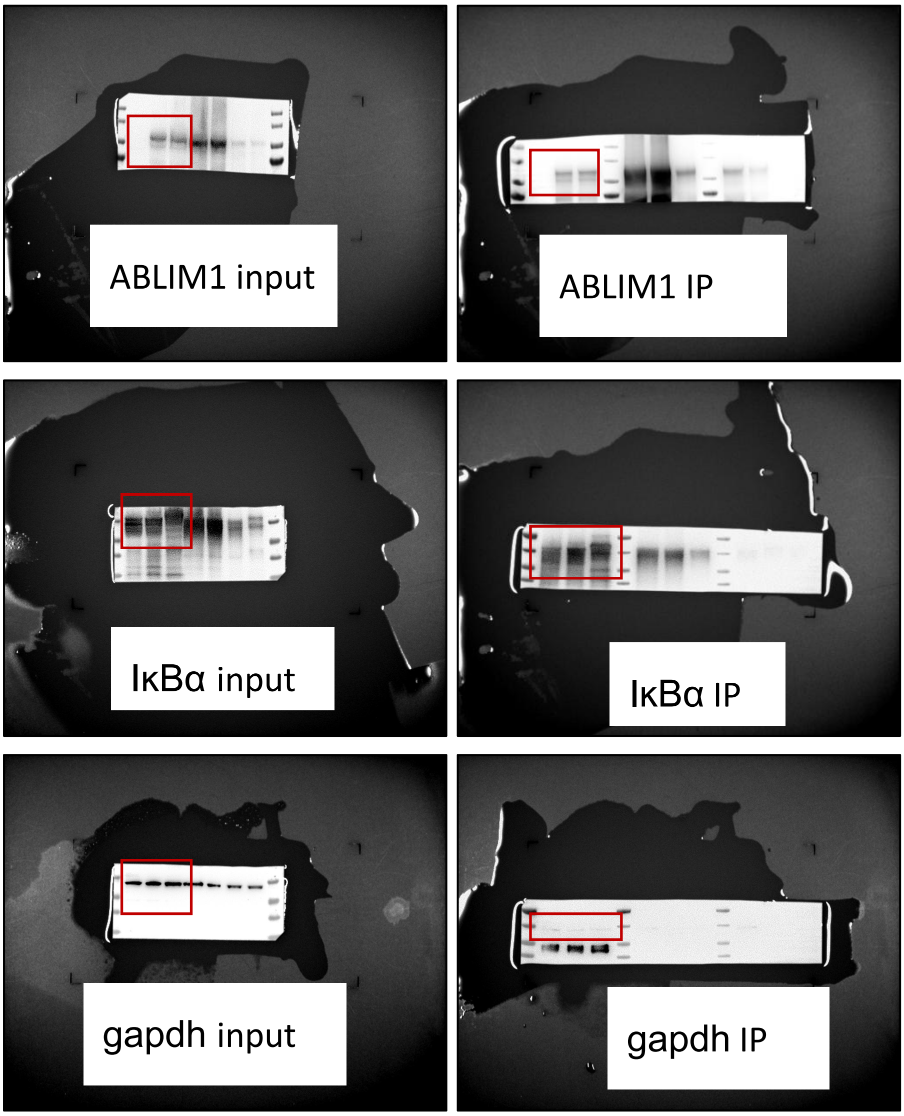
ABLIM1 input
ABLIM1 IP
IκBα input
IκBα IP
gapdh input
gapdh IP

Figure S4C

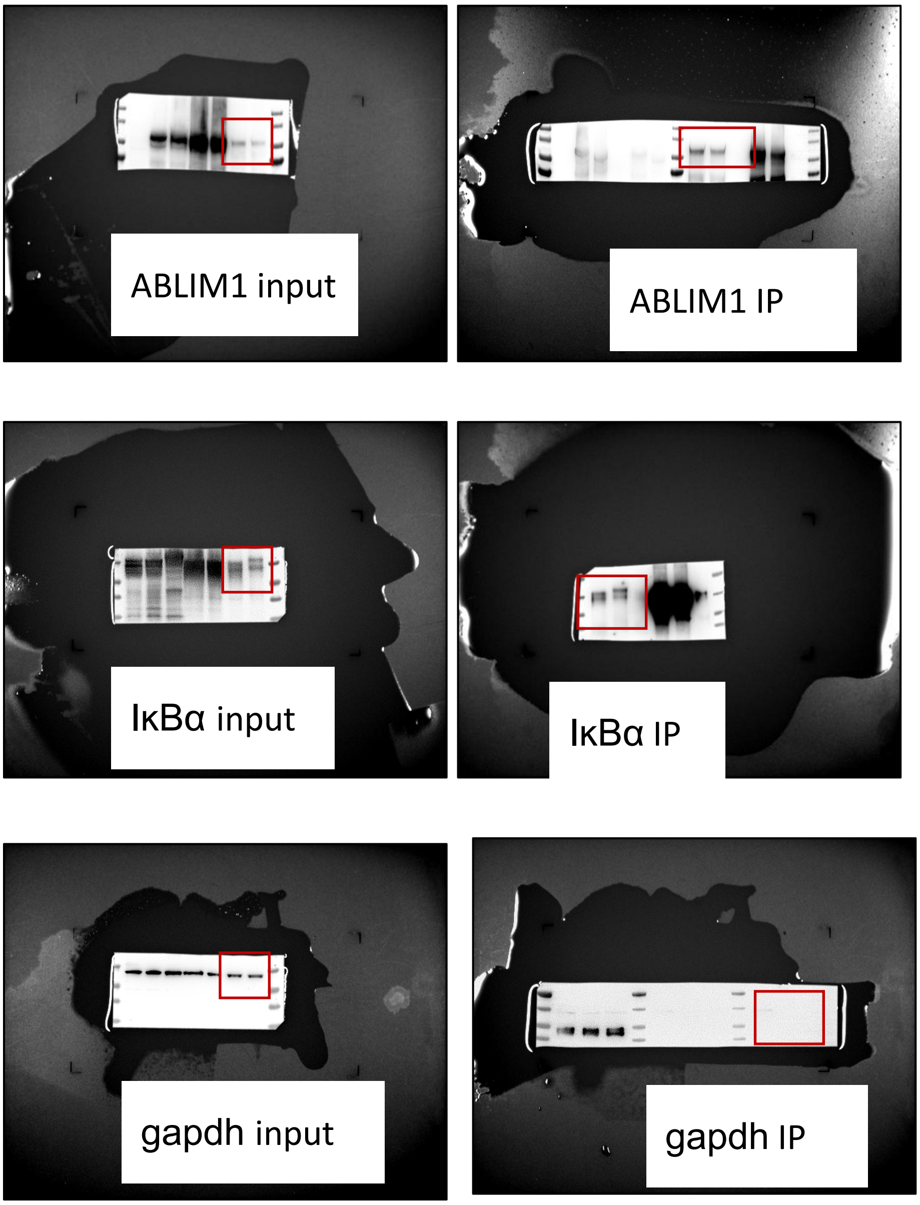
ABLIM1 input
ABLIM1 IP
IκBα input
IκBα IP
gapdh input
gapdh IP
